# Strain Control of Valley Polarization Dynamics in a 2D Semiconductor via Exciton Hybridization

**DOI:** 10.1021/acs.nanolett.5c02636

**Published:** 2025-10-13

**Authors:** Abhijeet M. Kumar, Douglas J. Bock, Denis Yagodkin, Edith Wietek, Bianca Höfer, Max Sinner, Adrián Dewambrechies, Pablo Hernández López, Sviatoslav Kovalchuk, Raghav Dhingra, Sebastian Heeg, Cornelius Gahl, Florian Libisch, Alexey Chernikov, Ermin Malic, Roberto Rosati, Kirill I. Bolotin

**Affiliations:** † Department of Physics, Freie Universität Berlin, Arnimallee 14, 14195 Berlin, Germany; ‡ Institute of Applied Physics and Würzburg-Dresden Cluster of Excellence ct.qmat, Technische Universität Dresden, 01062 Dresden, Germany; § Institute for Theoretical Physics, TU Wien, Wiedner Hauptstraße 8-10, 1040 Vienna, Austria; ∥ Department of Physics, Humboldt-Universität Berlin, Newtonstraße 15, 12489 Berlin, Germany and; ⊥ Department of Physics, Philipps-Universität Marburg, 35037 Marburg, Germany; ¶ Department of Physics and Materials Science, University of Luxembourg, L-1511 Luxembourg, EU, Luxembourg

**Keywords:** Valleytronics, Transition metal dichalcogenides (TMDs), 2D semiconductors, Spin/valley dynamics, Excitons, Mechanical strain

## Abstract

Encoding and manipulating digital information in quantum degrees of freedom is one of the major challenges of today’s science and technology. The valley indices of excitons in transition metal dichalcogenides (TMDs) are well-suited to addressing this challenge. Here, we employ mechanical strain to manipulate intervalley interactions and tune the valley polarization dynamics of excitons across a broader range of momentum space in monolayer TMDs. We use strain engineering to form valley-hybridized excitons that combine the advantages of bright intravalley excitons, where the valley index directly couples to light polarization, and dark intervalley excitons, characterized by low depolarization rates. We demonstrate that these valley-hybridized excitons exhibit signatures of coherently coupled states with a 100-fold reduction in valley depolarization rate and up to a 5-fold increase in steady-state valley polarization compared to previously studied excitons. Our findings of strain-tunable valley character of excitons advance the applications of TMDs in valleytronics.

## Graphical abstract

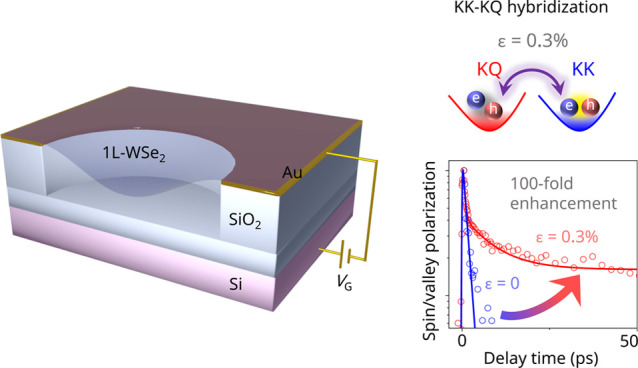

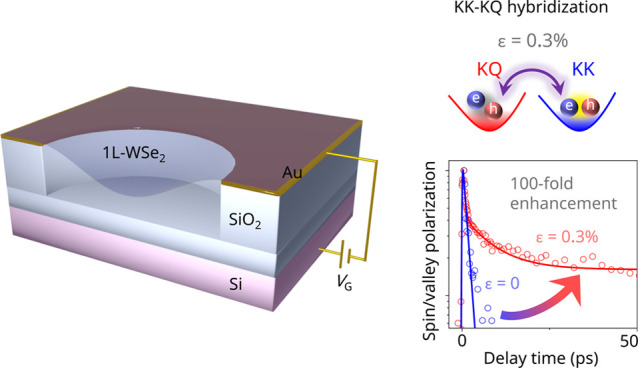

The emerging field of valleytronics aims to use the valley index of quasiparticles to store and process quantum information. Transition metal dichalcogenides (TMDs) from the family of layered 2D semiconductors are promising valleytronic materials, featuring energy-degenerate extremal points in their band structure (e.g., at K and K′ or Q and Q′) that host electronic wave functions. The K and K′ valleys can be selectively addressed by light chirality via valley-contrasting optical selection rules,^1,2^ with the bright energy-degenerate excitons, X_KK_ and X_K′K′_ (subscripts denote valley indices of hole and electron wave functions, respectively) inheriting the valley index.^3–6^ Experimental demonstrations of valley manipulation,^7–9^ coupling between valley and spin,^5,10,11^ and spatial transport^12–17^ have firmly established the potential of TMD excitons for valleytronics.

Complex interactions among various intra- and intervalley excitons drive the coupled spin and valley in TMDs.^12,18–100^ While the “bright” intravalley excitons X_KK_ and X_K′K′_ suffer rapid valley depolarization (∼a few picoseconds) at cryogenic temperatures,^24^ these excitons also interact with “dark” intervalley excitons, such as X_KQ_ or X_KK′_, that are protected from exchange-induced depolarization.^25,26^ These intervalley excitons are the lowest excited state in tungsten-based TMDs.^27–29^ Spin/valley coupling at the Q valley,^30^ similar to that at the K valley, adds further complexities to the dynamics associated with these states. Additionally, momentum-delocalized energy bands slightly below the conduction band, caused by lattice defects, influence valley depolarization dynamics by providing the necessary momentum for the radiative recombination of intervalley excitons.^31,32^ Despite highly suitable properties of the intervalley excitons for valleytronics, controlling interexcitonic interactions for tunable valley polarization dynamics in TMDs has remained largely unexplored.

Mechanical strain is a powerful tool to modulate intervalley interactions and tune the valley polarization dynamics in TMDs. Under an applied strain, different valleys exhibit distinct energy shifts, which manifest in the emission energies of associated excitons.^33,35^ At specific strain levels, a pair of bright and dark excitons with distinct valley character enter energetic resonance, giving rise to a new class of quasiparticles—valley-hybridized excitons.^32,34,36^ Crucially, valley-hybridized excitons present new means of controlling valley dynamics in TMDs, potentially surpassing conventionally probed bright and dark excitons. First, these excitons can inherit optical properties of their constituting states, such as a large oscillator strength and reduced intervalley exchange rate. Second, hybridization modifies intervalley interactions, enhancing control over dark excitons by mixing them with bright states. This effectively overcomes the usual limitations of conventional techniques capable of probing dark excitons—that require magnetic fields,^37^ surface plasmon polaritons,^38^ or waveguides.^39^ Finally, exciton hybridization can be externally tuned by controlling the strain state of the device. Despite the advantages of valley-hybridized excitons, their valleytronic properties have not been experimentally studied, to the best of our knowledge.

Here, we employ mechanical strain to tune valley polarization dynamics of excitons associated with K/K′ valley, Q valley, and the momentum-delocalized defect states in a monolayer of WSe_2_. First, for the hybridized states formed between X_KK′_ and defect-related excitons D^0^ near 0.8% strain, we observe up to a 5-fold increase in steady-state valley polarization and an increase in the radiative recombination rate compared to the dark excitons at zero strain. This observation is explained by the hybridization-induced lifting of momentum selection rules. Second, the hybridization between $X_{\mathrm{KK}}^{0}$ and X_KQ_ excitons at ∼0.3% strain exhibits a substantial increase in steady-state valley polarization and a striking 100-fold slowdown in depolarization dynamics, which we assign to the suppressed exchange interactions for the resulting coherent state.

## Strain Response of Excitons

Figure 1a shows a schematic band structure of unstrained 1L-WSe_2_ and selected excitonic transitions. The optically bright neutral excitons $X_{\mathrm{KK}}^{0}$ and $X_{K^{\prime }K^{\prime }}^{0}$ (blue) reside energetically above the dark intervalley excitons X_KK′_ (gray) and X_KQ_ (red). A defect-localized exciton D^0^ (green) is associated with momentum-delocalized energy bands arising from point defects, such as single selenium vacancies (Supporting Information Section S3). The radiative recombination of X_KK′_ and X_KQ_ requires a third particle (e.g., impurity, phonons, etc.).^28,40–43^ Since defects break translational symmetry, radiative recombination of D^0^ is allowed; however, its emission intensity is influenced by the defect density.

**Figure 1:**
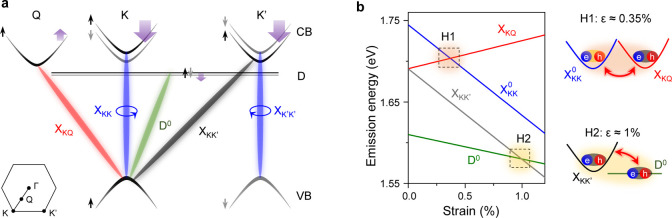
Exciton hybridization in 1L-WSe_2_. (a) Schematic band structure in 1L-WSe_2_ at zero strain with selected excitonic transitions: X_KK_ and X_K′K′_ (blue), X_KQ_ (red), X_KK′_ (gray), and D^0^ (green). X_KK_ and X_K′K′_ transitions couple to light with opposite chirality. The Q/Q′ valleys are also spin/valley locked, similar to the K/K′ valleys. The higher energy Q sub-band is not shown here due to a large spin-splitting (>100 meV). Purple arrows denote the strain response of valleys with respect to the valence band at the K valley. (b, left panel) Theoretically calculated strain response of excitons in 1L-WSe_2_ with same color-coding as in panel a. Reproduced in part with permission from our previous work, ref 34. Copyright 2024 Kumar et al. under CC-BY 4.0 license, publsihed by Springer Nature. Excitons from different valleys enter energetic resonance near specific strain values, denoted by shaded regions H1 and H2. Note that variations in defect geometry may shift their energies by a few tens of meV (Section S3). These calculations do not account for the influence of hybridization on energy shift. (b, right panel) Hybridized states of $X_{\mathrm{KK}}^{0}$ and X_KQ_ at ϵ ≈ 0.35% (H1, top), X_KK′_, and D^0^ at ϵ ≈ 1% (H2, bottom) in excitonic representation.

Under an applied mechanical strain, ϵ, different valleys exhibit contrasting energy shifts (purple arrows in Figure 1a), modifying the optical response of associated excitons.^31,33–35,44–48^ Figure 1b shows theoretically calculated exciton energies vs tensile biaxial strain in a 1L-WSe_2_. Near 0.35% strain, we observe dark X_KQ_ excitons entering energetic resonance with bright $X_{\mathrm{KK}}^{0}$ (region H1).^34^ Next, near 1% strain, intervalley excitons X_KK′_ become energy-resonant with D^0^ (region H2).^31,32^ In both cases, the resulting hybridized species acquire traits of the constituting excitons with different valley characters with the intervalley interactions being strain-dependent. Therefore, we expect strain-controlled exciton hybridization to strongly influence spin/valley dynamics in 1L-WSe_2_.

## Optical Detection of Valley-Hybridized Excitons

To induce mechanical strain, we employ an electrostatic gating-based straining technique.^32,34^ Here, a monolayer of WSe_2_ is suspended over a circular trench of diameter ∼ 5 μm in an Au/SiO_2_/Si substrate (Figure 2a). A gate voltage (*V*_G_) applied between WSe_2_ and Si deflects the membrane, inducing tensile biaxial strain in its center, which is symmetric vs the polarity of *V*_G_. Note that although carrier density is inherently linked to our straining approach, the effects of strain on excitonic energies can be distinguished from those originating from carrier density changes (Section S6).^32,34^ We record the excitons’ photoluminescence (PL) response at *T* = 10 K under CW laser excitation at 1.84 eV (Figure 2b; Section S1). In addition to the well-known bright excitons, we identify a series of dark intervalley excitons at ϵ = 0, including intervalley trions ( $X_{{\mathrm{KK}}^{\prime }}^{-}$ ) near 1.685 eV, their phonon replicas ( $X_{{\mathrm{KK}}^{\prime }\left(\gamma \right)}^{-}$ ) around 1.65–1.67 eV, and defect-related excitons near 1.60 eV by comparing their energetic positions and power dependence with previous reports.^32,34,49,50^

**Figure 2:**
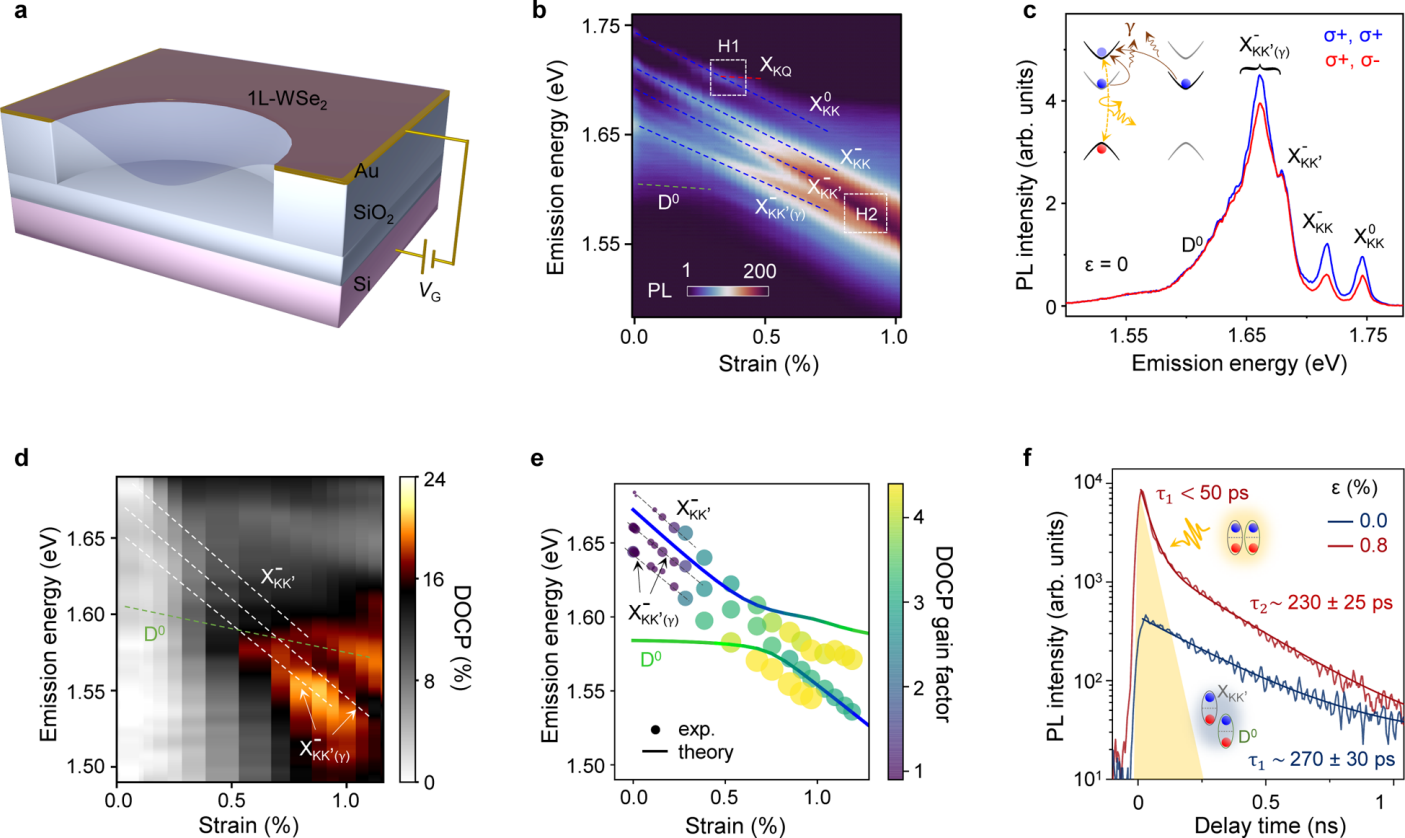
Strain tuning of dark K/K′ valley exciton polarization. (a) Schematic of the electrostatic straining technique. An applied gate voltage *V*_G_ induces biaxial strain in the center of the membrane. (b) PL vs strain false color map in 1L-WSe_2_ (device 1) at *T* = 10 K. The energy shifts of excitons and the hybridization regimes are labeled. (c) Co- and cross-polarized PL spectra (blue and red, respectively) from 1L-WSe_2_ at ϵ = 0 under σ+ excitation (see Section S6 for discussions on the influence of doping-related effects on DOCP). The inset shows radiative emission channels for dark trions $X_{{\mathrm{KK}}^{\prime }}^{-}$ mediated by chiral phonons (brown arrows). (d) DOCP vs strain false color map in 1L-WSe_2_ (acquired in a different *V*_G_ sweep than in panel b) for the states below 1.70 eV. White dashed lines denote the energy shift of $X_{{\mathrm{KK}}^{\prime }}^{-}$ and its two phonon replicas; the green line extrapolates the same for D^0^. (e) Scatter plot for the emission energy vs strain for $X_{{\mathrm{KK}}^{\prime }}^{-}$ and its phonon replicas; the area of the circle is proportional to the DOCP, the DOCP gain factor (η) for each state is color-coded. Theoretically calculated strain response of $X_{{\mathrm{KK}}^{\prime }}^{-}$ and D^0^ (solid lines), accounting for hybridization effects, reveal an avoided-crossing pattern; color of the line corresponds to the valley character of each exciton (blue: $X_{{\mathrm{KK}}^{\prime }}^{-}$ , green: D^0^), both lines are downshifted by 15 meV for clarity. (f) Time-resolved PL traces in the energetic vicinity of $X_{{\mathrm{KK}}^{\prime }}^{-}$ at zero strain (blue) and 0.8% strain (red) from the same device. PL intensity at *t* = 0 increases and a new decay component emerges at 0.8% strain, highlighted by yellow-shaded region; cartoons depict relative energetic alignment of X_KK′_ and D^0^ excitons.

Upon applying strain, we observed substantial changes in PL intensity and emission energies of excitons (Figure 2b). By comparing the extracted emission energies vs applied strain with the theoretical predictions in Figure 1b, we identify two hybridization regimes H1 and H2. First, near 0.35% strain, the dark excitons X_KQ_ brighten through hybridization with $X_{\mathrm{KK}}^{0}$ , exhibiting a distinct optical signature with the emission energy being strain-independent (see Section S2 and Figures S4 and  S9 for details).^34^ Second, near 0.8% strain, the intervalley excitons X_KK′_ hybridize with D^0^, leading to more than an order of magnitude increase in their PL intensity compared to the unstrained state (Figure 2b).^31,32^ The properties of these hybridized states are detailed in our recent reports.^32,34^

To probe steady-state exciton valley polarization, we employed polarization-resolved PL under circular and linear excitation, recording the degree of circular polarization (DOCP) and the degree of linear polarization (DOLP), respectively (Section S1). While DOCP quantifies the retention of an exciton’s valley memory over its lifetime, DOLP provides information about the quantum coherence of the entangled states across the two valleys.^8^ We note that unlike the case of bright neutral ( $X_{\mathrm{KK}}^{0}$ ) and charged ( $X_{\mathrm{KK}}^{+/-}$ ) excitons, the valley-selective optical selection rules do not directly apply to the intervalley and defect-related transitions. Nevertheless, previous works have demonstrated that the experimentally measured DOCP reliably characterizes their valley polarization state,^39,51^ forming the basis to probe the valley polarization of individual states in our device. Conversely, DOLP is nonzero only for $X_{\mathrm{KK}}^{0}$ (Figure S9). Valley coherence for dark- and many-body excitons is suppressed due to processes such as intervalley scattering^8^ and long lifetimes compared to $X_{\mathrm{KK}}^{0}$ .^52^

## Polarization Control of Hybridized X_KK′_–D^0^ Excitons

Having demonstrated strain-controlled exciton hybridization, we now investigate the valley-polarized response of K/K′ intervalley excitons as they enter energetic resonance with D^0^ (region H2 in Figures 1b and 2b). Figure 2c shows co- and cross-circularly polarized PL in 1L-WSe_2_ at zero strain. A vanishing DOCP for $X_{{\mathrm{KK}}^{\prime }}^{-}$ is consistent with their out-of-plane transition dipoles.^50,53,54^ A small DOCP < 5% observed from $X_{{\mathrm{KK}}^{\prime }\left(\gamma \right)}^{-}$ is likely associated with interactions involving chiral phonons (inset of Figure 2c),^50^ impurities,^49^ or resident carriers.^55^ No DOCP is observed from defect-related states near 1.60 eV. However, the DOCP for these states changes drastically under strain (Figure 2d). We quantify these changes by plotting the emission energy vs strain for selected dark states $X_{{\mathrm{KK}}^{\prime }}^{-}$ and $X_{{\mathrm{KK}}^{\prime }\left(\gamma \right)}^{-}$ , obtained using the fitting procedure described in ref 34, in Figure 2e; the color of the data points reflects the DOCP gain factor η, defined as $\eta =\frac{\mathrm{DOCP}\left(\epsilon \right)}{\mathrm{DOCP}\left(\epsilon =0\right)}$ . An experimental uncertainty in DOCP of 5% is assumed to evaluate η for states with vanishing DOCP at zero strain.

We make the following observations from the data in Figure 2e. First, a maximum in DOCP (η between 3 and 5) for dark trions and their phonon replicas is reached at state-specific strain values between 0.7 and 1%, coinciding with the point of hybridization of these states with D^0^. This behavior mimics the PL intensity of the same states, reaching its maximum in the same strain regime (Figure 2b). Second, the slope of the strain-dependent energy shift for the highest energy dark trions changes for ϵ > 1%, aligning with the trend of D^0^ excitons. This suggests that the dark trions acquire, at least partially, the character of D^0^, yet, surprisingly, they retain their valley polarization. This change in the valley character is confirmed by our first-principle calculations that show an avoided crossing behavior between the two states (solid lines in Figure 2e) by accounting for strain-dependent coupling between them (see Section S3 and Figures S3 and S5 for details). Finally, the temporal dynamics of the dark trions, recorded via time-resolved PL (see Section S1 for details), change significantly near the hybridization point (Figure 2f). At ϵ = 0.8%, the PL intensity at *t* = 0 increases 10-fold, accompanied by the emergence of a new decay component (τ_1_ < 50 ps), indicating a similar increase in the radiative recombination rate compared to the zero-strain case.

The data in Figure 2d–f collectively highlight the influence of interexcitonic hybridization on the valley polarization dynamics of dark K/K′ valley excitons. The amplification in DOCP—concomitant with a maximum in PL intensity, reduced radiative lifetime, and an avoided crossing pattern—is consistent with defects providing necessary momentum to the dark states for radiative emission under hybridization. The valley polarization is further strengthened by the hole’s valley state in the valence band, which predominantly resides in the valley of excitation. Notably, the D^0^ exciton retains valley polarization up to the maximum applied strain level of 1.8% (Figure S5), indicating that this state has inherited the valley character of “free” K/K′ valley excitons after hybridization. Finally, we note that the observed amplification in DOCP cannot be explained by changes in the carrier density in our devices (see Section S6 and Figures S6 and S7 for details).

## Polarization Control of Hybridized X^0^_KK_–X_KQ_ Excitons

Next, we focus on the hybridized state of X_KQ_ and $X_{\mathrm{KK}}^{0}$ excitons (region H1 in Figures 1b and 2b), characterized by a new peak in PL near 1.70 eV and near 0.35% strain (Figures S4 and S9).^34^ Panels a and b of Figure 3 show false color maps of DOCP and DOLP vs strain, respectively, in the energetic vicinity of $X_{\mathrm{KK}}^{0}$ . Both quantities increase in the strain regime corresponding to $X_{\mathrm{KK}}^{0}$ –X_KQ_ hybridization. Since the retention of linear polarization is a measure of exciton valley coherence, our observations suggest enhanced coupling between the two valleys and formation of a coherently coupled resonant hybridized state.

**Figure 3:**
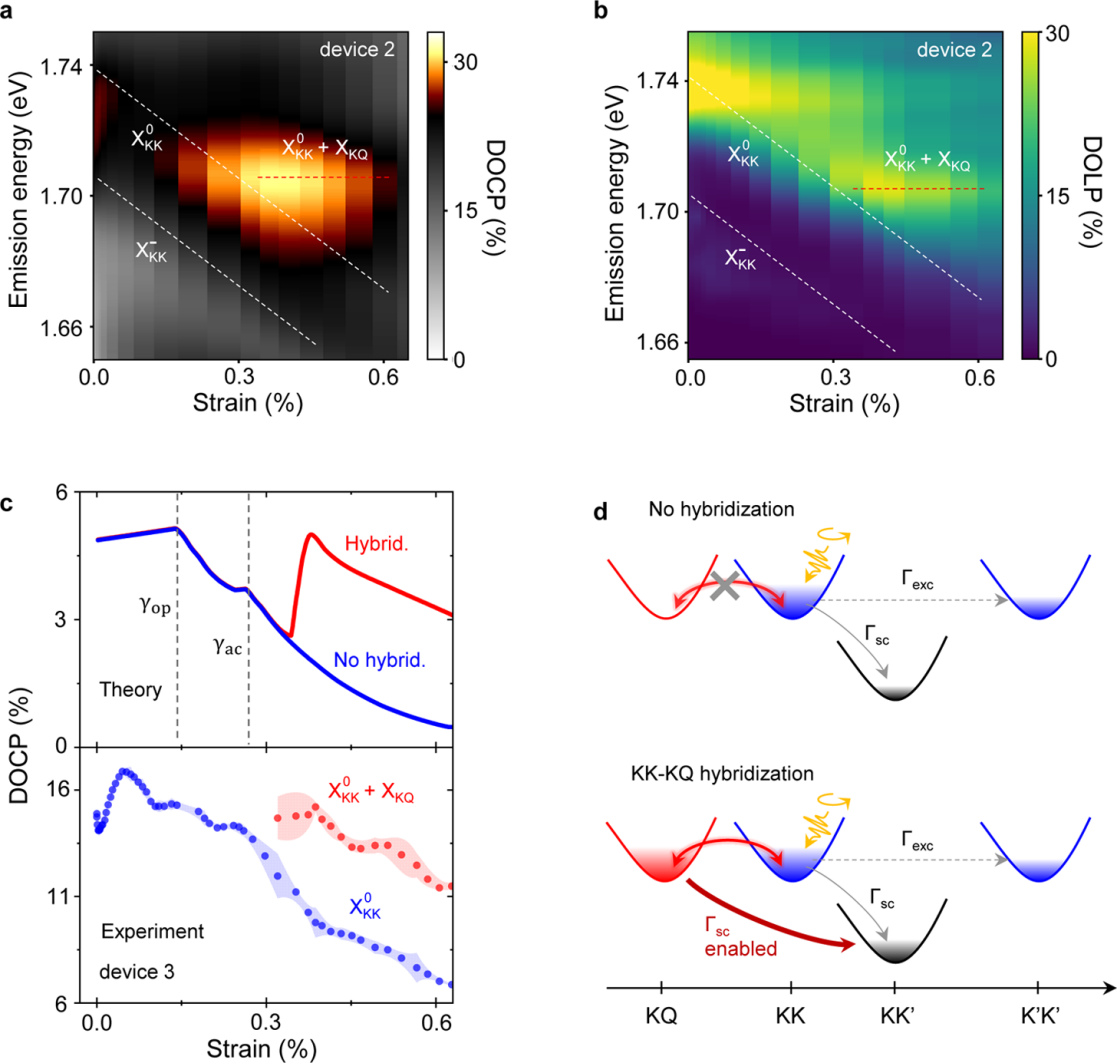
Valley polarization control by tuning KK–KQ coupling. (a, b) False color map of DOCP (a) and DOLP (b) vs strain from device 2. A local maximum in DOCP and DOLP is observed near 0.4% strain, suggesting an enhanced intervalley coupling and formation of a coherent hybridized state. Note, the nonzero polarization on the higher energy tail arises from excitons located away from the membrane center due to strain inhomogeneity. (c, top panel) Theoretically calculated DOCP vs strain for $X_{\mathrm{KK}}^{0}$ without (blue) and with (red) hybridization with X_KQ_. The two dashed lines denote strain values corresponding to the closing of optical (γ_op_)- and acoustic phonon (γ_ac_)-mediated scattering channel between $X_{\mathrm{KK}}^{0}$ and X_KQ_. (c, bottom panel) Experimentally obtained DOCP vs strain (from device 3). An increase in DOCP near 0.4% strain is observed for the hybridized state (red), showing qualitative agreement with the theory. The DOCP trend below 0.1% strain is not yet understood. The difference in the absolute DOCP magnitude between theory and experiments arises due to the variation in doping level in realistic devices.^56^ (d) Schematic representation of intervalley exciton scattering near ϵ = 0.35%. $X_{\mathrm{KK}}^{0}$ , $X_{K^{\prime }K^{\prime }}^{0}$ , and X_KQ_ are energy-resonant, lying above X_KK′_. Yellow arrows denote the valley-polarized excitation of KK excitons, whereas gray arrows denote intervalley exchange (dashed) and phonon-assisted scattering (solid). In the absence of hybridization (top), phonon-assisted scattering between $X_{\mathrm{KK}}^{0}$ and X_KQ_ is blocked. The hybridization (bottom) forms a resonantly coupled KK–KQ state and enables a new scattering channel between X_KQ_ and X_KK′_ (dark red arrow).

To explain our observations, we developed a many-particle theory to microscopically determine the strain-dependent exciton landscape, both with and without exciton hybridization. Starting from exciton dynamics in the presence of exchange interactions^26^ and doping,^56^ we obtain valley-resolved occupations of bright excitons under continuous-wave excitation (Section S2). We obtain

1 $$\mathrm{DOCP}=\frac{\Gamma_{\mathrm{dec}}}{2\Gamma_{\mathrm{exc}}+\Gamma_{\mathrm{dec}}}$$

where Γ_dec_ = Γ_rad_ + Γ_sc_ is the combined rates of the radiative decay of excitons (Γ_rad_) and phonon-assisted intervalley scattering (Γ_sc_), while excluding the intervalley exchange rate Γ_exc_. Note that the DOCP expression in eq 1 is consistent with an independently employed rate equation model.^5,57^ In the absence of hybridization, Γ_exc_ is microscopically evaluated as $\Gamma_{\mathrm{exc}}=\frac{2}{\Gamma_{\mathrm{sc}}}J^{2}$ , where *J* ^2^ = Σ_***q***_ |*J*_***q***_|^2^ *N*°_***q***_ is the squared modulus of the exchange Hamiltonian |*J*_***q***_|^2^, summed over the exciton momentum ***q*** and weighted by the normalized excitonic distribution, here assumed to be thermalized (Section S2). In agreement with the generalized Maialle–Silva–Sham model,^58,59^ the derived Γ_exc_ is quadratic in the exchange Hamiltonian—which depends weakly on strain—and scales with the inverse of the scattering rates Γ_sc_. We further evaluate microscopically the dependence of Γ_sc_ on strain and hybridization. Our final calculations for the DOCP of $X_{\mathrm{KK}}^{0}$ , via eq 1, show strong strain-dependent behavior (Figure 3c, top panel).

In the absence of hybridization, DOCP decreases with strain, exhibiting two pronounced drops near 0.15 and 0.27% strain (dashed lines in Figure 3c, top), corresponding to the closure of optical and acoustic phonon-assisted scattering channels between $X_{\mathrm{KK}}^{0}$ and X_KQ_, respectively (Section S2). Consequently, the decay rate Γ_dec_ and hence DOCP lower. Notably, DOCP increases sharply near ϵ = 0.35% when the hybridization between $X_{\mathrm{KK}}^{0}$ and X_KQ_ is accounted for (red in Figure 3c, top). Thanks to this hybridization, the bright states acquire a KQ component and, subsequently, gain the scattering channel from KQ to KK′. This scattering mechanism is particularly effective in this strain regime, as demonstrated recently by strain-dependent diffusion studies.^60^ In contrast, the scattering rate between $X_{\mathrm{KK}}^{0}$ and X_KK′_ changes only weakly due to their similar strain-dependent energy shifts (Figure S2). Additionally, we assume negligible changes in Γ_rad_ of $X_{\mathrm{KK}}^{0}$ as its binding energy remains nearly unaffected in the studied strain range.^34^ Consequently, this new scattering channel between the hybridized states and X_KK′_ leads to the increase of Γ_dec_ (depicted by the cartoon in Figure 3d) and, with it, to a local maximum in DOCP at ∼0.4% strain.

Our experimental observations in Figure 3c (bottom panel) are in qualitative agreement with theoretical predictions. Furthermore, a gradual decrease of DOCP vs strain for the hybridized state in experiments is ascribed to the spatial strain inhomogeneity in our device (see Section S2 and Figure S1 for details).^34^

## Ultrafast Dynamics of Hybridized X^0^_KK_–X_KQ_ Excitons

To directly access the spin/valley dynamics stemming from the resonant coupling between K and Q valley excitons, we employed single-color time-resolved Kerr rotation (TRKR) spectroscopy (Figure 4a; see Section S1 for details). We chose the temperature *T* = 100 K to minimize the contributions from dark excitons,^22,61^ resident carriers,^23,62,63^ and trap states^23,64^ that are dominant at cryogenic temperatures and complicate the interpretation of the data (black in Figure 4b).

**Figure 4:**
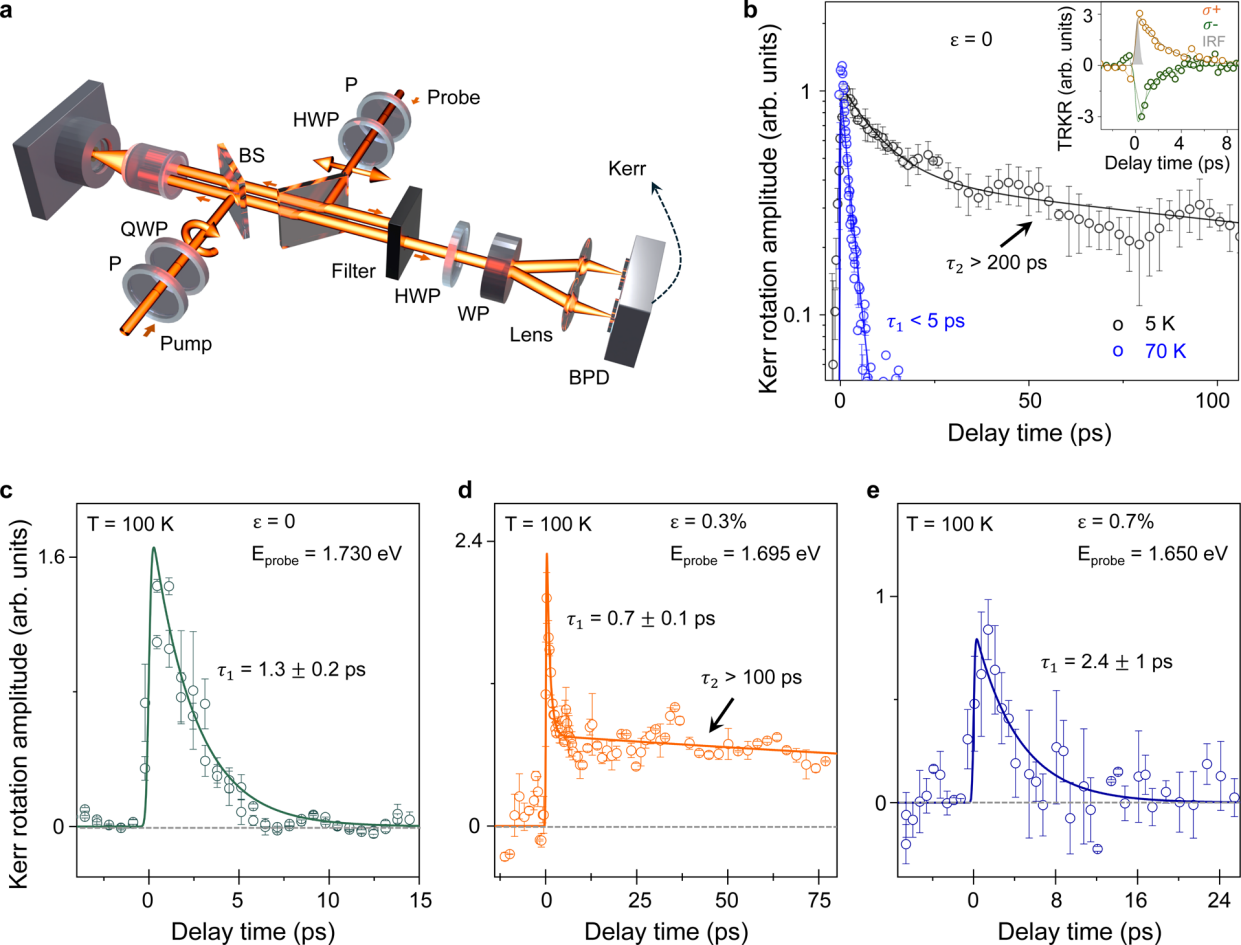
Ultrafast spin/valley dynamics in strained 1L-WSe_2_. (a) Schematic of single-color TRKR setup (P, polarizer; BS, beamsplitter; QWP, quarter-wave plate; HWP, half-wave plate; WP, Wollaston prism; BPD, balanced photodetector; see Section S1 for details). (b) Temperature-dependent TRKR dynamics in device 5 at zero strain (*E*_pump_ = *E*_probe_ = 1.72 eV). At *T* = 5 K (black), a second long-lived decay component (τ_2_ > 200 ps) persists due to contributions from trap states and resident carriers. This component vanishes at *T* = 70 K (blue), indicating negligible influence of such effects at elevated temperatures. The inset shows representative TRKR traces vs pump helicity (orange and green, respectively) together with the instrument response function (gray). (c–e) TRKR dynamics in device 2 at ϵ = 0, 0.3, and 0.7%, respectively, at *T* = 100 K. The pump and probe energies were set near the neutral exciton resonance for each strain value (1.730, 1.695, and 1.650 eV, respectively), determined via in situ PL measurements. Solid lines are fits; dashed gray lines correspond to zero TRKR amplitude. In the dynamics at ϵ = 0.3%, a second decay component with a time constant exceeding 100 ps is observed.

Panels c–e of Figure 4 show TRKR traces probed at $X_{\mathrm{KK}}^{0}$ resonance for selected strain values; solid lines are the fits to the data. We observe a rapid decay of the TRKR signal at zero strain (Figure 4c, τ < 2 ps), which can be explained by rapid exchange-mediated intervalley scattering—expected to be dominant under resonant pump excitation^65^—and phonon-mediated valley depolarization.^66^ In contrast, at ϵ = 0.3% (Figure 4d), the TRKR decay dynamics turn biexponential, with the second decay component (τ_2_) longer-lived compared to the unstrained case by more than 2 orders of magnitude. We ascribe this behavior to the KK state acquiring partial intervalley character through resonant coupling with the KQ state, for which the exchange scattering mechanism is suppressed.^26^ Correspondingly, the second decay component reflects a low rate of valley depolarization for the hybridized population. This assignment is further supported by a similar trend consistently recorded across devices with varying doping levels (see Section S8, Figures S11 and S12 for details). Additionally, the TRKR dynamics probed near the $X_{\mathrm{KK}}^{+/-}$ resonance do not exhibit any long-lived component across the applied gate voltage range (Figures S10–S12), ruling out our observations being governed by doping-related resident carrier depolarization.^23,62,63^ Furthermore, the spin/valley dynamics in our devices are free from substrate-related disorder, which is typically linked to doping-dependent transitions between mono- and biexponential decay profiles.^64^ Finally, the disappearance of the long-lived component in the TRKR dynamics at 0.7% strain (Figure 4e) is consistent with $X_{\mathrm{KK}}^{0}$ and X_KQ_ coming out of energetic resonance, suppressing the contribution of the X_KQ_ population to the observed Kerr signal. Overall, our observations in Figure 4 demonstrate the potential of strain in tuning ultrafast spin-valley dynamics in TMDs.

To summarize, we reported the first spectroscopic demonstration of strain-controlled intervalley coupling on valley polarization dynamics in WSe_2_ monolayers. We recorded a more than 3-fold increase in DOCP of dark K/K′ valley excitons upon hybridization with defect-related excitons. The hybridization with the KQ intervalley excitons increases the steady-state valley polarization of $X_{\mathrm{KK}}^{0}$ excitons and reduces their depolarization rates by more than 2 orders of magnitude.

Our findings open multiple directions for future research. First, future experiments with tunable, near-resonant excitation may achieve near 100% valley polarization of hybridized excitons. The reported valley polarization in our PL experiments is underestimated by up to 50% due to strain-induced energy detuning under fixed excitation energy (Figure S8). Long lifetimes of species associated with the Q valley may be utilized for spin-/valley-polarized transport, achievable in devices with engineered strain gradients. Second, our results open new paths for theoretical exploration to understand microscopic interactions between excitons and phonons in the presence of defects and the spin/valley depolarization mechanisms for the hybridized excitons. Finally, exploring the coherent nature of KK–KQ excitons under pseudomagnetic fields—arising from uniaxial strain^56,67–70^ —would be an interesting next step toward valley manipulation.

## Supplementary Material



## Data Availability

The data that support the findings of this study have been deposited in the Zenodo repository under the accession code: zenodo.org/records/16298712. Additional data may be obtained from the corresponding author upon reasonable request.

## Notes

(Section S1) Methods; (Section S2) microscopic many-particle modeling; (Section S3) strain response of defect excitons; (Section S4) extended note on KK-KQ hybridization; (Section S5) valley-polarized nature of defect excitons; (Section S6) extended analysis of doping effects in DOCP data; (Section S7) potential artifacts in DOCP data; (Section S8) extended discussion on TRKR data (PDF)
