# Supplementary material for: Strain Control of Valley Polarization Dynamics in a 2D Semiconductor via Exciton Hybridization
Source: Nano Lett. 2025 Oct 13;25(42):15164–72. doi: 10.1021/acs.nanolett.5c02636 (PMC12550856; doi:10.1021/acs.nanolett.5c02636)
Supplement: Supplementary file 1 [file nl5c02636_si_001.pdf]

Supporting Information
Strain control of valley polarization dynamics in a 2D semiconductor via exciton hybridization

Abhijeet M. Kumar[1], Douglas J. Bock[1], Denis Yagodkin[1], Edith Wietek[2], Bianca Höfer[1], Max Sinner[3], Adrián Dewambrechies[1], Pablo Hernández López[4], Sviatoslav Kovalchuk[1], Raghav Dhingra[1], Sebastian Heeg[4], Cornelius Gahl[1], Florian Libisch[3], Alexey Chernikov[2], Ermin Malic[5], Roberto Rosati[5], and Kirill I. Bolotin[1*]

[1]*Department of Physics, Freie Universität Berlin, Arnimallee 14, 14195 Berlin, Germany*
[2]*Institute of Applied Physics and Würzburg-Dresden Cluster of Excellence ct.qmat, Technische Universität Dresden, 01062 Dresden, Germany*
[3]*Institute for Theoretical Physics, TU Wien, Wiedner Hauptstraße 8-10, 1040 Vienna, Austria*
[4]*Department of Physics, Humboldt-Universität Berlin, Newtonstraße 15, 12489 Berlin, Germany and*
[5]*Department of Physics, Philipps-Universität Marburg, 35037 Marburg, Germany*

Contents

S1. Methods 2
S2. Microscopic many-particle Modeling 3
S3. Strain response of defect excitons 7
S4. Extended note on KK-KQ hybridization 9
S5. Valley-polarized nature of defect excitons 10
S6. Extended analysis of doping effects in DOCP data 10
S7. Potential artefacts in DOCP data 13
S8. Extended discussion on TRKR data 15
References 18

* kirill.bolotin@fu-berlin.de

S1 Methods

Sample fabrication: The WSe_2 flakes were mechanically exfoliated and transferred onto a circular trench (diameter is ∼5 µm) in a $Au/Cr/SiO_2/Si$ stack using a dry transfer approach. The cavity was developed via a wet etching process using Hydrofluoric (HF) acid. A gate voltage (typically in the range of up to ±210 V) was applied between the TMD flake (electrically grounded) and the Si back gate of the chip to induce strain. The strain in the center was characterized following the laser interferometry approach used in our recent work [1].

Polarization-resolved PL measurements: The devices were measured inside a cryostat (CryoVac Konti Micro) at a base temperature of 10 K. Devices 1 and 2 (corresponding to data in Figs. 2 and 3a,b of the main manuscript) were probed under a CW laser excitation at $\lambda = 670$ nm (6 µW), tightly focused in the center of the membrane using an objective (Olympus LMPlanFL N 50x/0.50) with spot diameter ∼1 µm. For device 3 (data in Fig. 3c of the main manuscript), we used a CW laser with $\lambda = 685$ nm and 2 µW power. A combination of a polarizer (GL 10, Thorlabs) and a quarter-wave plate (RAC 4.4.10, B. Halle) was used to control the circular polarization of excitation. For linearly-polarized excitation, the quarter-wave plate was replaced with a half-wave plate (RAC 4.2.10, B. Halle). Both wave plates were positioned before the objective, ensuring that the emitted light passed through them before being detected. To filter the polarization state of the emitted light, we used a combination of a half-wave plate and an analyzer before the spectrometer. The PL signal was detected using the Spectrometer, Kymera 193i Spectrograph.

The degree of circular polarization is quantified as, $\mathrm{DOCP} = \frac{I_{\mathrm{co}} - I_{\mathrm{cross}}}{I_{\mathrm{co}} + I_{\mathrm{cross}}}$, where I_{co} and I_{cross} are the co- and cross-polarized PL intensities under circularly polarized excitation, respectively. Conversely, the degree of linear polarization is measured as, $\mathrm{DOLP} = \frac{I_{\mathrm{H}} - I_{\mathrm{V}}}{I_{\mathrm{H}} + I_{\mathrm{V}}}$, where I_{H} and I_{V} are the two orthogonal linearly polarized PL components.

Time-resolved PL measurements: The sample was excited by a pulsed Ti:sapphire laser (Coherent Chameleon Ultra II, 140 fs pulse duration and 80 MHz repetition rate) at $\lambda = 700$ nm and 0.55 $\mu\mathrm{Jcm}^{-2}$ fluence, with a laser spot of ∼1 µm diameter, focused onto the sample using a 60x objective. The injected electron-hole pair density was on the order of $10^{10}\,\mathrm{cm}^{-2}$, where Auger-like exciton-exciton annihilation effects are negligible. A streak camera (C10910, Hamamatsu) was used to time- and spectrally resolve the PL signal.

Time-resolved Kerr rotation spectroscopy: We used a wavelength-tunable pulsed Ti:sapphire laser (Coherent Chameleon Ultra II, 140 fs pulse duration and 80 MHz repetition rate). The laser pulse was split into the pump and probe components (setup schematic in Fig. 4a in the main manuscript). Circular polarization of the pump pulse was controlled using a combination of a linear polarizer (GL 10, Thorlabs) and a quarter-wave plate (RAC 4.4.10, B. Halle). The pump and probe pulses were spatially separated but made collinear before being focused onto the sample at near-normal incidence using a reflective objective (LMM40X-P01, Thorlabs). The beam sizes were adjusted independently using a pair of telescopes to prevent clipping at the objective entrance. The pump and probe spot sizes were ∼3 and ∼1 µm, respectively. The reflected pump beam was blocked using a spatial filter. Extra care was taken to minimize any residual pump leakage onto the photodetector. The reflected linearly-polarized probe was separated into two components of orthogonal polarization after passing through an optical bridge consisting of a half-wave plate (RAC 4.2.10, B. Halle) and a Wollaston prism (PWK 10.10, B. Halle). Both polarization components were simultaneously recorded by using a home-built balanced photodetector, and the differential signal was read out using a lock-in amplifier phase-locked to a chopper in the pump path. For all TRKR measurements, two traces with opposite pump helicity were recorded sequentially, and the average of these traces was calculated. This ensured that any time-resolved signal that was helicity-independent canceled out. This step becomes especially important at higher strain values, where a finite membrane curvature could influence the light polarization upon reflection. Additionally, laser interference at zero delay obscures our ability to resolve the signal within the first 200–300 fs after photoexcitation. The strain level and corresponding exciton resonances during the TRKR measurements were characterized by *in-situ* PL (see Note S8 for details).

Microscopic many-particle modeling: We calculate exciton energies by solving a strain-dependent Wannier equation [1, 2], including the Keldysh-Rytova Coulomb potential [3, 4], and single-particle valley-dependent masses and strain-induced energy shifts [5–7]. The resulting excitonic eigenstates allow to evaluate the corresponding valley-dependent exciton-phonon scattering rates [2]. Assuming that hybridization occurs for quasi-resonant KK and KQ states, we are able to predict experimentally measured strain-dependent PL signatures. In order to predict the DOCP, we evaluate the relative occupation of bright exciton states with opposite circular polarization. For this purpose, we start from exciton dynamics in the presence of exchange interaction [8] and doping [9] and include the continuous-wave excitation. More details can be found in Note S2.

S2 Microscopic many-particle Modeling

Exciton strain response: We start by evaluating the strain-dependent excitonic energies, cf. Fig. 1b in the main manuscript. These are obtained by investigating homogeneous lattice deformations. First, we evaluate microscopically the excitonic energies by solving the Wannier equation with a generalized Rytova-Keldysh potential [3, 4, 10, 11] starting from the unstrained single-particle dispersion relation [5]. We then include the strain-induced effective mass variations [6] and spectral shifts [7], including a 10 meV smaller energy of the K valley in the conduction band to match the energy with the experiments [1], as justified by the uncertainty in the energy separation between K and Q valleys [5] as well for the spin-orbit splitting [12, 13]. We find well distinguishable fingerprints of the excitonic valley according to the strain-induced variation of their energy [1].

Exciton hybridization: We consider the bright states $|\mathrm{KK}\rangle$ as well as the three-fold degenerate states $|\mathrm{KQ}_i\rangle$, with $i = 1..3$ and with the respective energies $E^{\epsilon}_{\mathrm{KK}}$ and $E^{\epsilon}_{\mathrm{KQ}}$ that are determined as function of strain ϵ. In analogy to what has been done in Ref. [1], we assume that these two sets of states can hybridize with each other as

$$|\mathrm{KK}\rangle, |\mathrm{KQ}\rangle \to |\alpha\rangle = c^{\alpha}_{\mathrm{KK}}|\mathrm{KK}\rangle + \sum_i c^{\alpha}_{\mathrm{KQ}_i}|\mathrm{KQ}_i\rangle, \tag{S1}$$

resulting in the new hybridized states $|\alpha\rangle$ labeled by the index $\alpha = 0..3$. These correspond to a linear combination of the pristine excitons $|v\rangle$ with $v = \mathrm{KK}, \mathrm{KQ}_i$ weighted by the complex coefficients c^{α}_{v} fulfilling $\sum_v c^{\alpha *}_v c^{\alpha'}_v = \delta_{\alpha,\alpha'}$ [14]. The unhybridized states can be rewritten as a linear superposition of the hybridized states as

$$|v\rangle = \sum_{\alpha} d^{\alpha}_v |\alpha\rangle, \tag{S2}$$

with the coefficients d^{α}_v fulfilling completeness relation $\sum_{\alpha} d^{\alpha *}_v d^{\alpha}_{v'} = \delta_{v,v'}$. We take

$$|c^0_{\mathrm{KK}}|^2 = 1 - A^2_{\epsilon}, \quad |c^0_{\mathrm{KQ}_i}|^2 = \frac{1}{3}A^2_{\epsilon} \tag{S3a}$$

$$|c^n_{\mathrm{KK}}|^2 = A^2_{\epsilon}, \quad |c^n_{\mathrm{KQ}_i}|^2 = \frac{1}{3}\left(1 - A^2_{\epsilon}\right). \tag{S3b}$$

with n = 1,2,3, and with analogous assumptions for $|d^{\alpha}_{\mathrm{v}}|^2$, where A^2_{ϵ}

$$A^2_{\epsilon} = A^2_0 e^{-\frac{\left(E^{\epsilon}_{\mathrm{KK}} - E^{\epsilon}_{\mathrm{KQ}}\right)^2}{2\Delta^2_E}}, \tag{S4}$$

describes the hybridization induced by the strain ϵ. The amplitude A_0 corresponds to the maximum mixing between the excitonic states. Besides providing the correct normalization of the involved states via $\sum_v |c^{\alpha}_v|^2 = \sum_{\alpha} |d^{\alpha}_v|^2 = 1$, Eqs. (S3),(S4) imply that the mixing between $|\mathrm{KK}\rangle$ and $|\mathrm{KQ}\rangle$ takes place only when KK and KQ become quasi-degenerate thanks to the strain-induced spectral shifts, i.e. $E^{\epsilon}_{\mathrm{KK}} \approx E^{\epsilon}_{\mathrm{KQ}}$. In the opposite limit of $|E^{\epsilon}_{\mathrm{KK}} - E^{\epsilon}_{\mathrm{KQ}}| \gg \Delta_E$ one has $A_{\epsilon} \approx 0$, recovering the uncoupled regime $|\alpha = 0\rangle \approx |\mathrm{KK}\rangle$ and $|\alpha = 1, 2, 3\rangle$ as a combination of $|\mathrm{KQ}_i\rangle$. A similar assumption has made it possible to theoretically understand the strain-induced new peak appearing in the photoluminescence [1]. Here, and in the main manuscript, we take a realistic value of $A^2_0 = 0.4$ and Δ_E corresponding to a FWHM of 10 meV of the Gaussian in Eq. (S4).

Exchange-induced bright exciton occupations: In order to understand how strain affects the DOCP, we investigate the occupation of the bright excitons with different spins s = $\uparrow\uparrow, \downarrow\downarrow$, with the arrows referring to the spin of electron and hole constituting the exciton. We first start by neglecting the hybridization effects and start from the dynamics provided in Ref. [8] , which can be rewritten as (neglecting the strain label)

$$\partial_t N^s_{v;\boldsymbol{Q}} = \frac{1}{\hbar}\sum_{\boldsymbol{q}\in lc}\left[\Gamma_{\mathrm{KK}\to v;\boldsymbol{Q}}|P^s_{\boldsymbol{q}}|^2 - \gamma_{\mathrm{rad}}\delta_{\boldsymbol{q},\boldsymbol{Q}}N^s_{v;\boldsymbol{Q}}\right] + \frac{\partial N^s_{v'\boldsymbol{Q}}}{\partial t}\Big|_{scat} + \frac{2}{\hbar}Im(J^s_{\boldsymbol{Q}}C^{ss'}_{\boldsymbol{Q}})(1-\delta_{s,s'}) \quad , \tag{S5}$$

$$\partial_t C^{ss'}_{\boldsymbol{Q}} = \partial_t C^{ss'}_{\boldsymbol{Q}}\Big|_{scat} + \frac{1}{i\hbar} J^{s*}_{\boldsymbol{Q}} \left(N^{s}_{v_b,\boldsymbol{Q}} - N^{s'}_{v_b,\boldsymbol{Q}} \right) \quad . \tag{S6}$$

Here, we have introduced the incoherent densities $N^s_{v,\boldsymbol{Q}} = \langle \hat{X}^{s\dagger}_{v,\boldsymbol{Q}} \hat{X}^{s}_{v,\boldsymbol{Q}} \rangle$ of excitons with momentum $\boldsymbol{Q}$ in the valley v and with the spin $s =\uparrow\uparrow, \downarrow\downarrow$, where $\hat{X}^{s}_{v,\boldsymbol{Q}}$ and $\hat{X}^{s\dagger}_{v,\boldsymbol{Q}}$ are the corresponding exciton annihilation and creation operators. Additionally, we have introduced the coherent exciton density $P^s_{\boldsymbol{Q}} = \langle \hat{X}^{s}_{v_b,\boldsymbol{Q}} \rangle$ excited only for $\boldsymbol{Q}$ in the lightcone (lc) and intervalley coherence $C^{ss'}_{\boldsymbol{Q}} = \langle \hat{X}^{s\dagger}_{v_b,\boldsymbol{Q}} \hat{X}^{s'}_{v_b,\boldsymbol{Q}} \rangle$, where v_b indicates the bright valley, specifically $v_b = \mathrm{KK}$ and $v_b = \mathrm{K'K'}$ for $s =\uparrow\uparrow$ and $s =\downarrow\downarrow$, respectively. Furthermore, γ_{rad} and $\Gamma_{\mathrm{KK}\to v}$ provide the radiative rate and the spin-conserving scattering rates from KK states to valley v, respectively. Both quantities are identical for the two spins (for example, $\Gamma_{\mathrm{KK}\to\mathrm{KQ}} = \Gamma_{\mathrm{K'K'}\to\mathrm{K'Q'}}$). In the case of a polarized excitation, e.g., only $P^{\uparrow\uparrow}_{\mathbf{q}}$ is excited directly, while $P^{\downarrow\downarrow}_{\mathbf{q}}$ is formed as a second-order process from the optically excited $P^{\uparrow\uparrow}_{\mathbf{q}}$ and hence negligible [8]. Finally, $J^s_{\boldsymbol{Q}}$ is the coefficient of the exchange Hamiltonian, which reads $J^{\uparrow\uparrow}_{\boldsymbol{Q}} = -J_Q e^{-2i\theta_{\boldsymbol{Q}}} = J^{\downarrow\downarrow *}_{\boldsymbol{Q}}$, where $\theta_{\boldsymbol{Q}}$ is the in-plane angle of the momentum $\boldsymbol{Q}$ with modulus Q, while for J_Q we take $J_Q = J\frac{Q^2}{K(Q+Q_{TF})}$, with $J = 1\,\mathrm{eV}$, as well as $\mathrm{K} = 1.26\,\text{Å}^{-1}$, and $Q_{\mathrm{TF}} = \frac{10 E^{\epsilon=0}_{\mathrm{KK}}}{\hbar c}$ to include doping of approximately $10^{11}\,\mathrm{cm}^{-2}$ [9].

Now, we consider the ratio $\frac{N^{\uparrow\uparrow}_{\mathrm{K'K'};0}}{N^{\downarrow\downarrow}_{\mathrm{KK};0}}$ between the occupation of bright states within the light cone for the two spin configurations. For this purpose, without loss of generality, we consider a circularly-polarized continuous-wave excitation exciting only $P^{\uparrow\uparrow}_{\mathbf{q}}$ and study the steady-state densities $N^{\downarrow\downarrow}_{\mathrm{cw;K'K'}}$ and $N^{\uparrow\uparrow}_{\mathrm{cw;KK}}$, fulfilling the condition $\partial_t N^{\downarrow\downarrow}_{\mathrm{cw;K'K'}} = 0$. It is convenient to express

$$N^s_{v;\boldsymbol{Q}} = N^{s^\circ}_{v;\boldsymbol{Q}} + \delta N^{s^\circ}_{v;\boldsymbol{Q}}$$

$$C^{ss'}_{\boldsymbol{Q}} = C^{ss'^\circ}_{\boldsymbol{Q}} + \delta C^{ss'}_{\boldsymbol{Q}},$$

where $\delta N^s_{v;\boldsymbol{Q}}$ indicates how much $N^s_{v;Q}$ differs from the thermalized Boltzmann distribution $N^{s^\circ}_{v;\boldsymbol{Q}}$, and analogously for $C^{ss'}_{\boldsymbol{Q}}$. Making use of the typical approximation for the incoming term $\partial_t \delta C^{ss'}_{\boldsymbol{Q}}\big|_{\text{scat - in-term}} \approx 0$, similarly to the one leading to the relaxation-time approximation[15], in the static limit we obtain

$$\delta C^{ss'}_{\mathrm{cw};\boldsymbol{Q}} = \frac{J^{s*}_{\boldsymbol{Q}}}{i\Gamma_{\mathrm{sc}}} \left(\delta N^{s}_{v_b,\boldsymbol{Q}} - \delta N^{s'}_{v_b,\boldsymbol{Q}} \right) \equiv \frac{J^{s^*}_{\boldsymbol{Q}}}{i\Gamma_{\mathrm{sc}}} \left(N^{s}_{v_b,\boldsymbol{Q}} - N^{s'}_{v_b,\boldsymbol{Q}} \right), \tag{S7}$$

where we used that $N^{s^\circ}_{v_{b,s},\boldsymbol{Q}} = N^{s'^\circ}_{v_{b,s'},\boldsymbol{Q}}$, and that only terms with $s \neq s'$ are relevant due to the factor $(1-\delta_{s,s'})$. Inserting this result into the dynamics of $N^{\uparrow\uparrow}_{\mathrm{K'K'};\boldsymbol{Q}}$ in the static limit we find

$$\frac{N^{\downarrow\downarrow}_{\mathrm{cw;K'K'}}}{N^{\uparrow\uparrow}_{\mathrm{cw;KK}}} \approx \frac{\frac{2}{\Gamma_{\mathrm{sc}}} \left\langle |J^{\downarrow\downarrow}_{Q}|^2 \right\rangle_{N^{\downarrow\downarrow}}}{(\gamma^{\mathrm{eff}}_{\mathrm{rad}} + \Gamma_{\mathrm{sc}}) + \frac{2}{\Gamma_{\mathrm{sc}}} \left\langle |J^{\downarrow\downarrow}_{Q}|^2 \right\rangle_{N^{\downarrow\downarrow}}}, \tag{S8}$$

where $\Gamma_{\mathrm{sc}} = \sum_{v,\boldsymbol{Q}} \Gamma_{\mathrm{KK},0\to v;\boldsymbol{Q}} \equiv \Gamma_{\mathrm{KK}} = \sum_{v,Q} \Gamma_{\mathrm{K'K'}\to v;\boldsymbol{Q}}$, $\gamma^{\mathrm{eff}}_{\mathrm{rad}} = \frac{\sum_{\mathbf{q}\in \mathrm{lc}} \gamma_{\mathrm{rad}} N^s_{v;\mathbf{q}}}{N^s_{v_{b,s}}}$, and $\left\langle |J^s_{\boldsymbol{Q}}|^2 \right\rangle_{N^s} = \frac{\sum_{\boldsymbol{Q}} |J^s_{\boldsymbol{Q}}|^2 N^s_{v_{(b,s)};\boldsymbol{Q}}}{N^s_{v_{b,s}}}$ with the assumption $\left(\Gamma_{\mathrm{sc}} |P^{\downarrow\downarrow}_{\mathrm{cw,K'K'}}|^2 + \sum_{v\neq \mathrm{K'K'}} \Gamma_{v\to \mathrm{K'K'},0} N^{\downarrow\downarrow}_{\mathrm{cw,K'K'},\boldsymbol{Q}} \right) \ll \frac{2}{\Gamma_{\mathrm{sc}}} \left\langle |J^{\downarrow\downarrow}_{\boldsymbol{Q}}|^2 \right\rangle_{N^{\downarrow\downarrow}}$ in view of the direct optical excitation $|P^{\uparrow\uparrow}_{\mathrm{cw,KK}}|^2$ and with $N^s_{v_{b,s}} = \sum_{\boldsymbol{Q}} N^s_{v_{b,s};\boldsymbol{Q}}$. Equation (S8) provides the ratio between bright excitons with the spin $\downarrow\downarrow$ compared to those with the spin $\uparrow\uparrow$ after a direct excitation of the latter. Similar to the generalized Maialle-Silva-Sham model [16, 17], it shows that the occupation of the $\downarrow\downarrow$ states depends quadratically on the efficiency of the exchange potential. When the latter is negligible, all excitons remain in the optically excited spin configuration. In the opposite limit, when the exchange potential is much larger than the scattering rate, the ratio between the two species approaches 1, meaning a negligible DOCP.

In analogy, this derivation can be extended to include hybridization. Focusing on the brightest state $|\alpha = 0\rangle$, we take

$$\frac{N^{\downarrow\downarrow}_{\mathrm{cw};\alpha=0}}{N^{\uparrow\uparrow}_{\mathrm{cw};\alpha=0}} \approx \frac{\sum_{\alpha'} \frac{2}{\Gamma_{0\alpha'}} \left\langle |J^{(0,\downarrow\downarrow)(\alpha'\uparrow\uparrow)}_{\boldsymbol{Q}}|^2 \right\rangle_{N^{\downarrow\downarrow}}}{\left(\gamma^{\alpha=0,\mathrm{eff}}_{\mathrm{rad}} + \Gamma_{\alpha=0}\right) + \sum_{\alpha'} \frac{2}{\Gamma_{0\alpha'}} \left\langle |J^{(0,\downarrow\downarrow)(\alpha'\uparrow\uparrow)}_{\boldsymbol{Q}}|^2 \right\rangle_{N^{\downarrow\downarrow}}}, \tag{S9}$$

with $J^{(\alpha,s)(\alpha' s')}_{\boldsymbol{Q}} = c^{\alpha *}_{v_b,s} c^{\alpha'}_{v_b,s'} J^{ss'}_{\boldsymbol{Q}}$, $\Gamma_{\alpha\alpha'} = \frac{\Gamma_\alpha + \Gamma_{\alpha'}}{2}$, and $\gamma^{(\alpha,\mathrm{eff})}_{\mathrm{rad}} = \left|c^{\alpha}_{v_b,s'}\right|^2 \gamma^{\mathrm{eff}}_{\mathrm{rad}}$ as the hybridization-induced variation of the average squared exchange potential, scattering rate, and effective radiative rate, respectively. Here we stress that the scattering rates $\Gamma_\alpha = \sum_v |c^\alpha_v|^2 \Gamma_v$ depend strongly on strain [1], crucially impacting the strain-dependent DOCP, as discussed below. Equation (S9) provides the ratio between bright excitons with the spin $\downarrow\downarrow$ compared to those with the spin $\uparrow\uparrow$ after a direct excitation of the latter through the knowledge of $|c^\alpha_{\mathrm{KK}}|^2$. This allows us to determine the DOCP as we discuss in the following.

Photoluminescence and DOCP: The knowledge of the spin-dependent bright-exciton occupation provided in Eqs. (S8) and (S9) for the cases without and with hybridization allows to determine the DOCP. Neglecting at first the strain, this can in general be rewritten as

$$\mathrm{DOCP} = \frac{I_{\mathrm{co}} - I_{\mathrm{cross}}}{I_{\mathrm{co}} + I_{\mathrm{cross}}} \equiv \frac{1 - \frac{N_{\mathrm{cross}}}{N_{\mathrm{co}}}}{1 + \frac{N_{\mathrm{cross}}}{N_{\mathrm{co}}}} = \frac{\Gamma_{\mathrm{dec}}}{\Gamma_{\mathrm{dec}} + 2\Gamma_{\mathrm{exc}}}, \tag{S10}$$

where, compared to Eq. (S9), $\frac{N_{\mathrm{cross}}}{N_{\mathrm{co}}}$ corresponds to $\frac{N^{\downarrow\downarrow}_{\mathrm{cw};\alpha=0}}{N^{\uparrow\uparrow}_{\mathrm{cw};\alpha=0}}$, while we have $\Gamma_{\mathrm{dec}} = \left(\gamma^{(\alpha=0,\mathrm{eff})}_{\mathrm{rad}} + \Gamma_{\alpha=0}\right)$, and $\Gamma_{\mathrm{exc}} = \sum_{\alpha'} \frac{2}{\Gamma_{0\alpha'}} \langle |J^{(0,\downarrow\downarrow)(\alpha'\uparrow\uparrow)}_{Q}|^2 \rangle$. To describe the situation in the experiment, we have to include strain as well as the inhomogeneity of the spatial profile of the exciton density. The interplay between position-dependent strain and exciton densities leads to peculiar effects in the space-integrated photoluminescence (PL), as shown in our previous study [1]. Here, we extend the procedure to describe the DOCP via Eqs. (S8)-(S10). In order to achieve this goal, we first introduce a Gaussian exciton and strain distribution $N(\mathbf{r})$ and $\epsilon(\mathbf{r})$,

$$N(\mathbf{r}) = N_0 e^{\frac{-r^2}{2\Delta_r^2}}, \quad \text{and} \quad \epsilon(\mathbf{r}) = \epsilon_0 e^{\frac{-r^2}{2\Delta_s^2}}, \tag{S11}$$

with N_0 and ϵ_0 being the maximum exciton density and strain, respectively. The exact value of N_0 has no impact on the spectral shape of the photoluminescence or the DOCP, as we are in the low exciton-density regime. In contrast, the maximum strain ϵ_0 crucially determines the DOCP and in general the optical response, first by changing the relative energy E^ϵ_v of different excitonic valleys v (cf. Fig. 1b of the main manuscript) and consequently by changing the phonon-driven scattering rates Γ_α. Furthermore, the shape of strain and the exciton spatial profile play a crucial role. We take the widths Δ_s and Δ_r as in our previous study [1], in particular corresponding to a FWHM of 6.1 µm and 2 µm, respectively. Such a shape of $s(\mathbf{r})$ agrees well with the experimental strain profile in the center of the sample (corresponding to $\epsilon(r = 1\,\mu\mathrm{m}) \approx 0.9 \cdot \epsilon_0$, cf. Sec. S7 and Fig. 3 in the main manuscript) [1]. We take an excitonic profile twice larger than the 1 µm laser spot, since the PL profile is typically larger than the laser width due to exciton diffusion and related propagation effects [18–20]. Due to the inhomogeneities in strain and exciton profile, the photoluminescence $I(\mathbf{r}, E)$ becomes space-dependent, reading

$$I(\mathbf{r}, E) \propto N_{br}(\mathbf{r}) I_{\epsilon(\mathbf{r})}(E), \tag{S12}$$

where $N_{br}(\mathbf{r})$ and $I_\epsilon(E)$ are the bright exciton density and the strain-dependent PL, respectively, and they read

$$N_{br}(\mathbf{r}) \equiv N_{br}(\epsilon(\mathbf{r})) = f_{br}(\epsilon(\mathbf{r})) N(\epsilon(\mathbf{r})) \quad \text{and} \tag{S13}$$

$$I_\epsilon(E) \propto \frac{\gamma(\gamma + \Gamma^\epsilon_{v_b}) h_\epsilon}{\left(E - E^\epsilon_{\mathrm{KK}}(\mathbf{r})\right)^2 + \left(\gamma + \Gamma^\epsilon_{v_b}\right)^2} \quad . \tag{S14}$$

The bright exciton density $N_{br}(\epsilon(\mathbf{r}))$ depends on the optically injected exciton density $N(\mathbf{r})$ times the fraction of the bright states $f_{br}(\epsilon)$, which, assuming a local equilibrium, depends on strain as $f_{br}(\epsilon) = e^{-\frac{E^\epsilon_{\mathrm{KK}}}{k_B T}} / \sum_{\boldsymbol{Q},v} g_v e^{-\frac{E^\epsilon_v + \frac{\hbar^2 Q^2}{2M_v}}{k_B T}}$, where g_v, M_v, and T are the valley degeneracy, the valley total mass, and the temperature, respectively. The spectrum

$I_\epsilon(E)$ describes the PL emitted around the energy $E^{\epsilon}_{\rm KK}$ via an Elliott formula [21] with the radiative-recombination rate γ [6] and the exciton-phonon scattering rates $\Gamma^{s}_{v_b}$ of bright excitons (evaluated microscopically as in Refs. [22, 23]). Finally, h_ϵ describes the strain-dependent exciton hybridization, reading $h_\epsilon = 1 + 2A^2_\epsilon > 1$ in view of the 3-fold degeneracy of KQ states, cf. Eq (S4). We recover the regular PL in the absence of hybridization ($A^2_\epsilon \to 0$). In this way, the new peak in the PL observed in a restricted range of strain has been theoretically predicted and attributed to KQ excitons [1].

Finally, the space-integrated PL is given by

$$I_{\rm 2D}(E) = \int d\mathbf{r} I(\mathbf{r}, E) \equiv \int dr I^{\{r\}}_{\rm 2D}(E), \quad \text{with} \quad I^{\{r\}}_{\rm 2D}(E) = 2\pi f_{\rm br}(\epsilon) N(\epsilon(r)) r I_{\epsilon(r)}(E), \tag{S15}$$

where we made use of the angular symmetry of both the exciton and the strain profile. Importantly, the presence of $r \equiv |\mathbf{r}|$ on the right-hand side of Eq. (S2) extends the range of emitting positions, contrary to the unrealistic case of one-dimensional cuts [1]. Equation (S15) provides the space-integrated intensity as the sum of the local PL and exciton density. Besides affecting the appearance of $X_{\rm KQ}$ [1], this has a crucial impact on the DOCP.

Generalizing Eq. (S10) to include inhomogeneous strain, we find the energy-dependent, space-integrated degree of circular polarization $\bar{D}(E)$ as

$$\bar{D}(E) = \frac{\int d\mathbf{r}\, I(\mathbf{r}, E)\, DOCP(\epsilon(\mathbf{r}))\, N(\epsilon(\mathbf{r}))}{\int d\mathbf{r}\, I(\mathbf{r}, E)\, N(\epsilon(\mathbf{r}))}, \tag{S16}$$

with $\mathrm{DOCP}(\epsilon(\mathbf{r})) = \frac{\Gamma_{\rm dec}(\epsilon(\mathbf{r}))}{\Gamma_{\rm dec}(\epsilon(\mathbf{r}))+2\Gamma_{\rm exc}(\epsilon(\mathbf{r}))}$ being the straight-forward generalization of the DOCP in Eq. (S10) after including the strain dependence of the scattering rates.

Eq. (S16) allows to reproduce the measured behaviour of polarization with strain, which crucially depends on the inhomogeneity of the strain profile. To show this, in Fig. S1 we compare the maximum value of the strain-dependent DOCP with and without inhomogeneity. In the first case, we take $\epsilon(\mathbf{r})$ as in Eq. (S11), whereas in the second case we take $\epsilon(\mathbf{r}) \equiv \epsilon_0$. The two results are identical at very small strain values, while differing at larger values. In particular, the results with inhomogeneous strain appear as a coarse-graining of the results with the homogeneous strain. This happens because an inhomogeneous strain profile leads to a spatially dependent $\mathrm{DOCP}(\epsilon) \equiv \mathrm{DOCP}(\epsilon(\mathbf{r}))$. While in the center of the membrane, the emitted PL shows the DOCP corresponding to the maximum strain ϵ_0, away from the center, the strain is smaller and results in a higher DOCP. As a consequence, in the presence of inhomogeneous strain, the DOCP is larger than in the center of the membrane. Importantly, this leads to the very slow decrease of DOCP with increasing strain predicted by our simulations with inhomogeneous strain and in excellent agreement with the experiments, cf. Fig. 3 of the main manuscript. Besides at very small strain, the results with and without

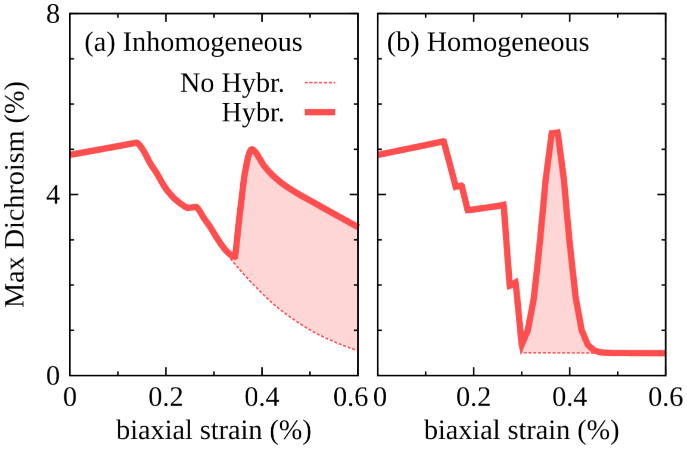

FIG. S1. **DOCP of hybridized KK-KQ state under inhomogeneous strain.** Circular polarization with (red) and without (blue) hybridization as a function of strain in the presence of (a) realistic, slightly inhomogeneous strain profile and (b) spatially homogeneous strain. When the strain is inhomogeneous, the PL emitted from different spatial points brings different DOCP, contrary to the case of homogeneous strain. As a consequence, the DOCP after spatial integration of inhomogeneous PL is a coarse-graining of the homogeneous case.

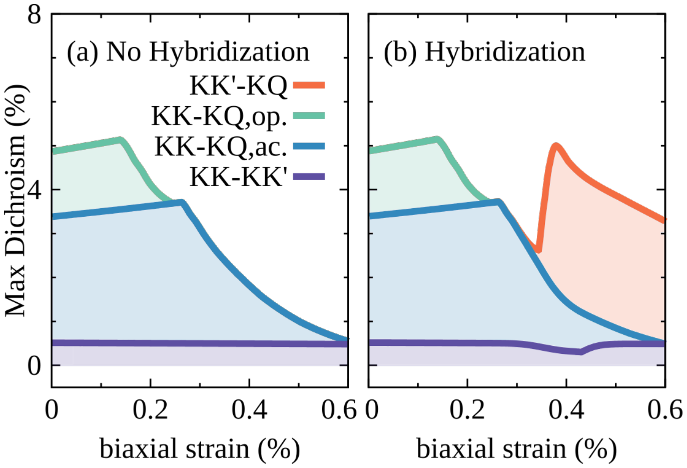

FIG. S2. **Contributions of different scattering channels to DOCP.** Individual contribution of different scattering channels to the DOCP (a) without and (b) with hybridization. Thanks to the three-fold degeneracy of the Q valley, the scattering from bright KK states into dark KQ excitons dominates the DOCP, and its strain-induced suppression is clearly visible as sharp decreases of DOCP. In the presence of hybridization, the bright states acquire a KQ component, hence gaining the new scattering channel from KQ to KK', which dominates the polarization at high strain values under hybridization (orange), while having no impact without hybridization.

inhomogeneous strain agree also with the case of high strain $\epsilon \geq 1.2\%$ (not shown).

Independent of the presence of hybridization, we observe two step-like decreases in the DOCP at $\epsilon \approx 0.15\,\%$ and $\epsilon \approx 0.27\,\%$, cf. Fig. S1(a,b). Similar to the case of the predicted linewidth [1], we can clearly associate these sharp variations to the closing of specific phonon-driven scattering channels. To show this, in Fig. S5 we separate the contribution of different scattering channels to the DOCP both without and with hybridization. The scattering between bright KK and KK' states is weakly affected by strain, because this changes only weakly the energy separation between these two states, in agreement with the measured energy position of spin-dark and bright states [24]. Such a scattering channel is, however, much less efficient than the one between KK and KQ states, in particular in view of the three-fold degeneracy of the latter. As a consequence, the DOCP is dominated by the scattering channel between KK and KQ states, which is strongly affected by strain, considering the opposite strain gauge factors of these two valleys. This results in a strain-induced closing of intervalley scattering, first via emission of optical phonons (energy of approximately 27–31 meV) and then of acoustic phonons (energy of approximately 15–18 meV; Ref. [25]). Such closing crucially affects the DOCP, resulting in two clear, sharp decreases when these scattering channels are suppressed, cf. green and blue lines in Fig. S2.

In the presence of hybridization, the bright states acquire a KQ component, which opens the scattering channel between KK' and KQ valley, which, thanks to the large deformation potential [25], is very efficient. This is reflected by the strain-dependent exciton diffusion coefficient, which at high temperatures increases by a factor of 3 with increasing strain, as recently shown in a joint theory-experiment study [2, 26]. In view of its efficiency, such scattering channels dominate the DOCP in the strain range, in which hybridization is allowed, cf. the orange line in Fig. S2.

S3 Strain response of defect excitons

To simulate the strain response of defect excitons, we calculate the strain-dependent hybridization between neutral donor states (D^0) and K/K' valley excitons in WSe_2 at selenium monovacancies. We first parametrize a strain-dependent tight-binding model from electronic structure calculations of WSe_2 at different strain values. A full tight-binding description containing all 2×11 (5d and $2 \times 3p$, $\times 2$ for spin) orbital basis functions of the outer shells in the WSe_2 unit cell. We use the VASP software package, including non-collinear spin calculations, to faithfully model spin-orbit coupling. We then extract maximally localized Wannier functions using Wannier90, derived from the W-5d and Se-4p orbitals, which provide a sufficiently robust basis for constructing the tight-binding Hamiltonian and accurately capture the band structure around the band gap at high symmetry points of interest (namely K/K').

To investigate the influence of strain, we implement a distance-dependent tight-binding model through a continuous

interpolation scheme based on five reference calculations at different strain configurations (-2 % to 2 % biaxial strain). Care must be taken to converge the Wannier projections at different strain values to compatible tight-binding descriptions. This approach allows us to track the evolution of electronic states while maintaining the physical constraints imposed by the crystal symmetry. The selenium monovacancy is modeled within a 12× 12 supercell — a size where the energy of the defect state becomes quite independent of k (as periodic defect-defect interactions become suppressed at the larger supercell. The removal of a selenium atom creates two localized defect states (see Fig. S3) whose character and energetic position depend sensitively on the local strain field. We refit the on-site matrix elements of the W orbitals around the vacancy to improve the agreement of the defect state energy with the experiment - indeed, the necessity of such a fit is expected for the standard exchange-correlation functionals that underestimate, e.g., the band gap of WSe_2.

We evaluate the band structure of the supercell as a function of strain, and find the expected crossing between the conduction band minimum and the defect state. Of particular interest is the interaction between these defect states and the conduction band minimum (CBM) of the host material. Our calculations reveal an avoided crossing between the vacancy-induced states and the CBM (at ∼0.8 % strain), characteristic of the hybridization between localized defect states and extended band states. This hybridization manifests in this mean-field description as avoided crossings between the constituent single-particle orbitals. In the excitonic picture, this crossing causes, at the right strain value, a mixing between the defect states and the K/K' valley excitons, providing a tunable platform for controlling the optical properties of the system. Fig. S3a shows the energy states in a single-particle picture. Note the small spin-orbit splitting in the defect states (compared to the conduction band), as well as the subsequent crossing with higher-lying conduction band states (labeled CB3 and CB4 in the picture). For even larger supercells, the energetic spacing to the next-higher conduction-band states decreases, turning the subsequent crossings between the defect state and the continuum of conduction-band states to a diabatic ridge [27]. We evaluate the excitonic energies to first order within the single-particle description as $E^{\text{exc.}}_{\text{approx.}} = E^{\text{def/band}}(\epsilon) - E^{\text{CBM}}(\epsilon = 0) + E^{\text{CBM}}(\epsilon)$, assuming a strain-independent exciton binding energy (Fig. S3b).

To check the polarization response of valley-hybridized states, we analyzed valley polarization for optical de-excitation of the defect state at a specific valley – the valley here being identified by the spin degree of freedom, as the flat defect band does not retain valley information. While we do find, as expected, that the hybridization prevents perfect valley polarization, we find a polarization of up to 80 % is retained via the spin degree of freedom.

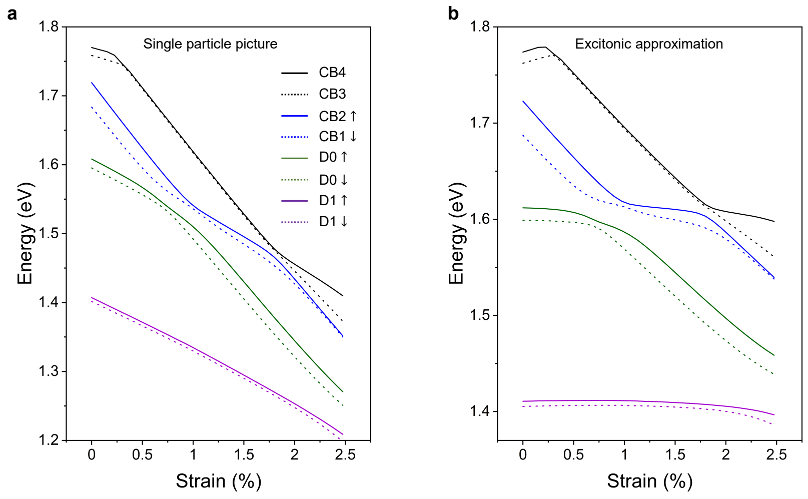

FIG. S3. **Theoretical calculations for KK'–D^0 hybridization.** Strain response of different energy levels accounting for the hybridization between defect energy bands and the conduction bands in single particle picture (**a**) and excitonic approximation (**b**). Two in-gap defect energy bands D^0 (green) and D^1 (purple), each with small spin-split sub-bands, are associated with the Se-vacancy point defects. Within the applied strain range in our studies, we are sensitive to the hybridization between D^0 (green) and the two conduction bands (blue). The higher energy bands CB3 and CB4 are the 'next' conduction band levels that appear due to back-folding of CB in the finite supercell used for our calculations. For an 'infinitely' large cell, these energy bands would be a continuum.

A note on the energy position of defects: In our picture of defect hybridization, we consider the point defects associated with a single chalcogen-vacancy in the WSe_2 crystal. However, this is a simplified approximation, especially in the presence of other point defects common to TMDs [28, 29]. Consequently, variations in energy dispersion and emission energy positions are expected that may alter the hybridization conditions. For example, the

data in devices 1 and 3 are consistent with defect hybridization near 1.59 ± 0.01 eV. In contrast, device 2, which was fabricated using a different batch of WSe_2 crystal, shows results consistent with hybridization near 1.62 ± 0.01 eV.

To account for the peak energy variations, we performed additional DFT calculations for slight variations of the defect geometry. In particular, we displaced one Se atom spatially close to the Se-vacancy into a metastable configuration or, alternatively, substituted one Se by O, then re-optimized the geometry (to the local minimum of this distortion). This resulted in slight variations of energy level alignments, ranging from 20 meV (one O-for-Se substitution of a Se atom in the Se layer opposite the one with the Se vacancy) to 110 meV (moving one of the Se atoms adjacent to the vacancy). Given the many possible configurations of displacing a few Se atoms, this easily accounts for the variation seen in the experiment, even not considering local charge modulations, etc..

S4 Extended note on KK-KQ hybridization

Under an applied biaxial strain, the Q valley shifts up in energy. Consequently, a KQ exciton becomes energy-resonant with the bright KK exciton near 0.3 % strain, resulting in its resonant brightening. A finite strain inhomogeneity in our devices turns this brightening condition a space-dependent phenomena: As the strain level in the membrane center exceeds 0.3 %, the resonant condition (i.e., $\epsilon = 0.3\,\%$) and, hence, the brightened emission of X_{KQ} shifts radially outward from the center, emitting at a constant energy where $E_{KK} = E_{KQ}$ [1]. Therefore, to resolve certain signatures of hybridization, such as avoided crossing, reducing the influence of strain inhomogeneity is crucial.

To this end, we modified our optical detection pathway by installing a pinhole system and an objective lens with a 100X magnification, which enabled us to collect a PL signal from a desired spatial location with ∼450 nm diameter. Figure S4a shows a finely-resolved false color PL map in the energetic vicinity of X^0_{KK} in device 5. The inset compares PL spectra with (solid white) and without a pinhole (dashed white): X_{KQ} shows higher PL intensity relative to X^0_{KK} when the signal is integrated across a larger spatial region, consistent with the inhomogeneity argument. Selected line cuts are plotted in Fig. S4b. We observe that a single peak X^0_{KK} is observed near 1.705 eV until ∼0.32 % strain. Interestingly, as the applied strain is increased by 0.01 % (expected shift in X^0_{KK} by ∼1 meV), we observe an emergence of a second peak on the lower energy side of X^0_{KK}. As the strain is increased further, the higher energy peak — the expected peak X^0_{KK} — is pinned near 1.704 eV, while the newly emerged lower energy peak progressively shifts down in energy, mimicking the strain response of X^0_{KK}. This observation is characterized by an energy splitting between the two peaks, with the higher energy state being X_{KQ}. We carefully fitted the data to resolve the minimum energy splitting between the two peaks, $\Delta E \approx 2.4 \pm 0.6$ meV. The corresponding coupling strength, $\Delta E/2$, is nearly an order of magnitude smaller than the excitonic linewidth, suggesting only moderate local KK-KQ coupling. In the future, devices with narrower linewidths (via hBN encapsulation) and an extension of theoretical modeling will further advance the understanding of the coherently coupled nature of these hybridized states.

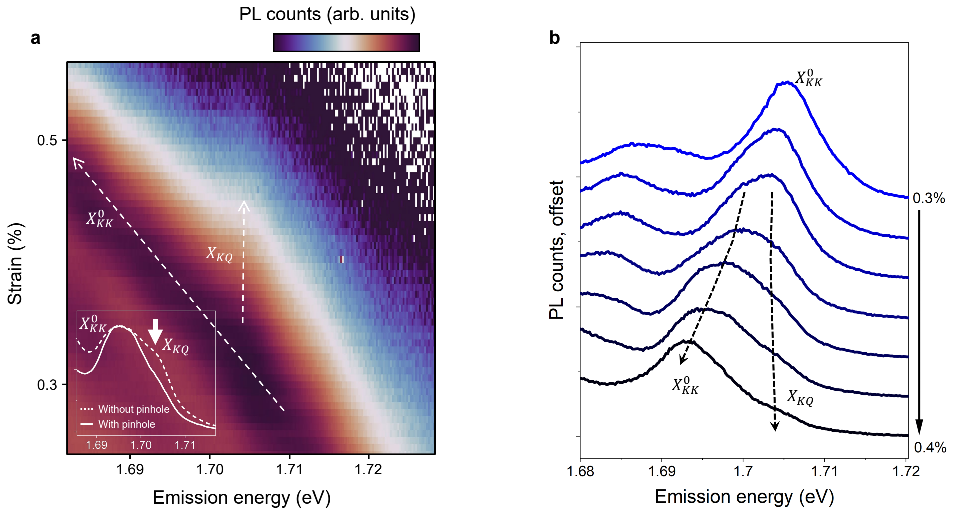

FIG. S4. **KK-KQ coupling.** **a** False color PL map vs. strain in vicinity of KK-KQ hybridization regime (device 5). The inset compares relative X^0_{KK} and X_{KQ} intensity at 0.38 % strain. **b** Selected line cuts reveal avoided-crossing-induced peak splitting (highlighted by dashed lines) and the evolution of X_{KQ}.

S5 Valley-polarized nature of defect excitons

Our data for device 1 (Fig. 2 of the main manuscript) reveal finite DOCP for the higher energy state near 1.58 eV above 1 % strain, as it spectrally separates from the K/K' intervalley excitons. A weak strain dependence of this state confirms its defect-like nature, while its optical visibility is strongly supported by the hybridization-induced increase in the oscillator strength. The valley-polarized emission of this state is particularly intriguing, as this state is optically accessible only through hybridization. This hybridization likely enables the state to inherit the valley character of 'free' K/K' valley excitons, opening the possibility to access its polarization properties. We note that the spin/valley properties of defect excitons are theoretically predicted due to finite contributions from their orbital and spin magnetic moments [30].

The observation of valley-polarized D^0 exciton was also reproduced in device 2 (Fig. S5a). In this device, the maximum applied strain reached 1.8 %, allowing a clearer identification of its valley-polarized nature. The strain response of this state is strongly supported by hybridization-induced avoided crossing (solid lines in Fig. S5b). Interestingly, this state retains higher valley polarization compared to the dark K/K' valley trions for $\epsilon > 1\,\%$, coinciding with the regime where this state is expected to couple with the bright excitons X^0_{KK}. While we did not observe a prominent optical signature of X^0_{KK} excitons in this strain range, the sustained valley polarization of D^0 exciton suggests it inherits the valley polarization of the bright KK excitons.

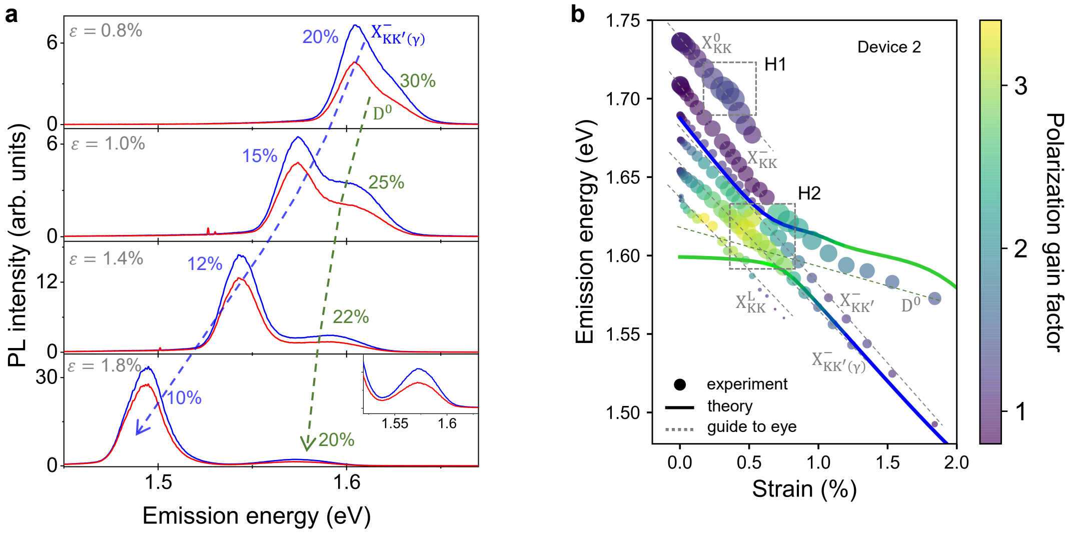

FIG. S5. **Polarization response of D^0 excitons in device 2.** **a** Co- (blue) and cross-circularly (red) polarized PL spectra at selected strain values from device 2. The D^0 state (green arrow) emerges on the higher energy side of dark trions (blue arrow) near 1 % strain, showing weaker strain response. Corresponding DOCP values for the two states are labeled and color-coded. Interestingly, D^0 maintains higher DOCP (inset) compared to the dark trions until the maximum applied strain level of 1.8 %. **b** Scatter plot for the emission energy vs. strain for different excitonic states from device 2, the DOCP gain factor (extracted using the same procedure as in Fig. 2e of the main manuscript) is color-coded; X^L_{KK} denotes defect-bound excitons [1]. Dashed lines are guides to the eyes, solid lines are theoretically calculated strain response of K/K' valley dark trions (blue) and D^0 (green), accounting for the hybridization effect. The strain response in energy shift is supported by the hybridization-induced avoided crossing, similar to the data presented in Fig. 2 in the main manuscript.

S6 Extended analysis of doping effects in DOCP data

Estimation of doping effects: In our straining approach, the applied V_G generates strain together with changes in the carrier density (n_e or n_h). The change in the carrier density in our suspended devices can be estimated using a plate capacitor model:

$$n_{e,h} = \frac{(V_G - V_0)\varepsilon_0}{e}\left(\frac{\varepsilon_{SiO_2}}{d_{SiO_2} + \varepsilon_{SiO_2}(d_{Au} - d(V_G))}\right) \tag{S17}$$

Here ε_0 and $\varepsilon_{\mathrm{SiO_2}} = 3.6$ are the vacuum permittivity and the dielectric constant of SiO_2, respectively. V_0 is the gate voltage at which the sample is charge neutral. $d_{\mathrm{SiO_2}} = 900\,\mathrm{nm}$ is the SiO_2 thickness, $d_{\mathrm{Au}} = 600\,\mathrm{nm}$ is the distance between the gold surface and SiO_2, $d(V_G)$ is the flake deflection (obtained from interferometry measurements) and e is the elemental charge. From this method, we obtain $n_{\mathrm{e,h}} \sim 0.8 \cdot 10^{12}\,\mathrm{cm}^{-2}$ at $V_G = -150\,\mathrm{V}$. This method accounts for changes in the capacitance under membrane deflection; however, its accuracy is limited by artefacts such as Schottky barriers, defects, photo-doping, etc., which introduce a nonlinear dependence of $n_{\mathrm{e,h}}$ on V_G. To validate the accuracy of this approach, we monitor the exciton-trion energy separation (ΔE_{XT}), which provides a precise measure of the Fermi energy shift, as $\Delta E_{\mathrm{XT}} \approx E_F$ [31]. The carrier density, in turn, relates to the Fermi energy as

$$n_{\mathrm{e,h}} = \frac{E_F m_{\mathrm{e,h}}}{\pi \hbar^2} \tag{S18}$$

Here, $m_{\mathrm{e,h}} = 0.36(0.40) \cdot m_0$ is the effective mass of an electron (hole) [5]. Equation (S18) estimates carrier density changes (n_e) on the order of $1 \cdot 10^{12}\,\mathrm{cm}^{-2}$ for 1 % applied strain [1].

Reproducibility across devices: The changes in DOCP observed in our data (Figs. 2 and 3 in the main manuscript) could also result from changes in the carrier density, and not the strain. To distinguish strain-related effects from doping, we recorded DOCP response to strain across three different devices (Fig. S6). The intrinsic doping level is different in each of these devices; red arrows in Fig. S6a-c, determined from gate-dependent neutral/charged exciton energy separation, indicate the electron (n_e) or hole (n_h) density in each device near 0.05 % strain. This means that different devices have different carrier densities when the strain level required for the hybridization be-

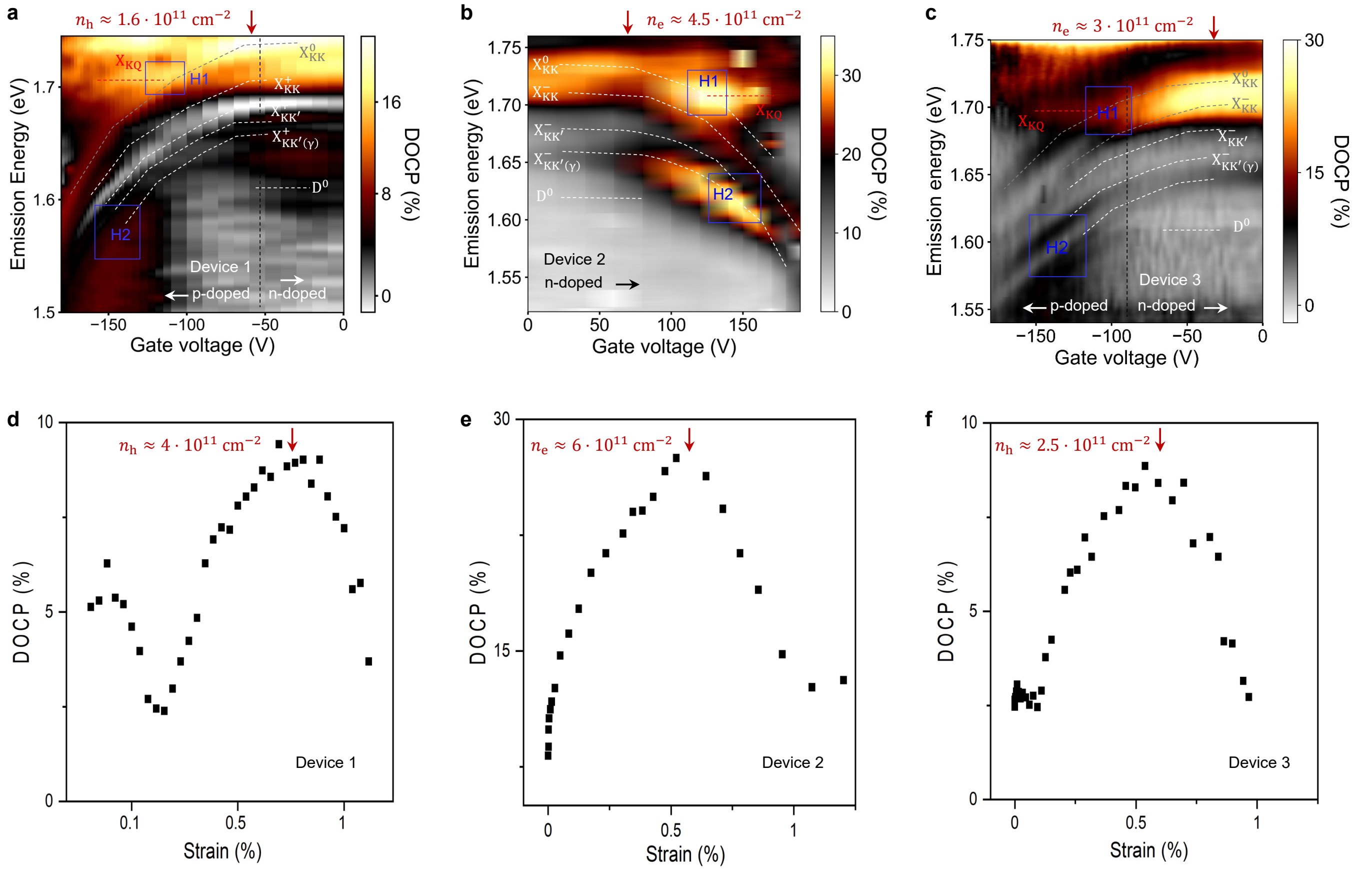

FIG. S6. **DOCP measurements across different devices. a-c** False color DOCP map vs. V_G in three different devices at $T = 10\,\mathrm{K}$. White and gray dashed lines are guides to the eyes for different excitonic states; black dashed lines, when present, denote the charge neutrality point. Enclosed regions (blue) highlight the two hybridization regimes. **d-f** Extracted strain dependence of DOCP for dark trions and their phonon replicas in respective devices. Each device has a different doping level at zero strain and near the point of hybridization (marked by red arrows). Device 1 (a) is the same as in Fig. 2 of the main manuscript, but p-doped at the point of hybridization under reversed V_G polarity. DOCP in devices 1 and 2 was measured under 1.84 eV CW excitation (6 μW power), while device 3 was excited using a 1.81 eV (2 μW) CW laser.

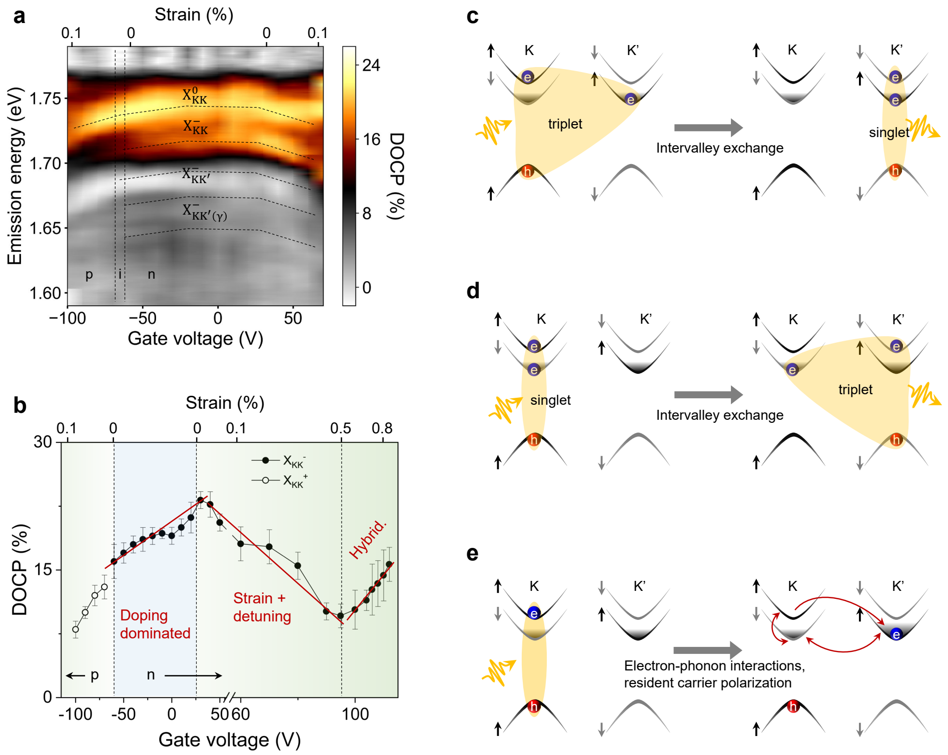

FIG. S7. **DOCP of bright trions.** **a** DOCP vs. V_G false color map in device 1 in the weak strain regime. **b** Extracted DOCP vs. V_G for the bright trions. The DOCP increases linearly in the doping-dominated regime (blue shaded region, $n_e = 1.5 \cdot 10^{11}\,\mathrm{cm}^{-2}$ near $V_G = +30\,\mathrm{V}$), but becomes non-monotonic under strain: it decreases until the strain level reaches a value $\sim 0.5\,\%$, before showing a slight increase towards 0.8 % strain. **c-d** Schematic of valley-polarized emission for triplet (c) and singlet (d) trions, exhibiting opposite circular polarization. **e** Schematic of inter- and intravalley scattering (red arrows) upon valley-polarized photoexcitation. The formation of singlet and triplet trions and their valley-polarized emission are sensitive to the resident electrons and the inter- and intra-valley scattering rates [33]. We speculate that the hybridization between the defects and the lower CB also changes the intervalley scattering rates and the DOCP for the bright trions, which is hinted in a slight increase in their DOCP towards 0.8 % strain.

tween KK' and D^0 excitons is reached. Consistent with expectations, we find a local maximum in DOCP for strain level between 0.6 and 0.8%. At the point of local DOCP maximum (with up to four-fold enhancement), each device has a different doping level (red arrows in Fig. S6d-f). Nevertheless, the absolute magnitude of DOCP is smaller in p-doped devices — a common feature of WSe_2 in doping-dependent polarization trend [32]. The DOCP decreases again as the applied strain is further increased and the states come out of hybridization. These observations confirm that while the carrier density-related effects are present, they do not cause the observed maximum in the DOCP trend.

Doping and strain response of bright trions: The bright K/K' valley trions X^-_{KK} involve an electron from the lower conduction sub-bands. While their valley polarization is significantly dependent on the doping level [33, 34], it may also be influenced by the hybridization between the CB and the defect energy bands. Figure S7a shows false-color DOCP map vs. V_G in the weak strain regime ($\sim 0.1\,\%$). The device is n-doped for $V_G > -60\,\mathrm{V}$; the doping-related effects dominate the strain-related effects until $V_G \approx +30\,\mathrm{V}$. The extracted DOCP for X^-_{KK} vs. V_G and strain in Fig. S7b show a complex behaviour. The DOCP for X^-_{KK} increases in the voltage regime, $V_G \in [-60, 25]\,\mathrm{V}$ (blue-shaded region), predominately arising from changes in the carrier density. In this regime, the Fermi energy level enters the CB, therefore, an increasing trend in DOCP is consistent with n-doped 1L-WSe_2 devices [34]. However, the interpretation of the data becomes challenging when strain-related effects become significant. First, a non-monotonic trend is observed around $+30\,\mathrm{V}$, accompanied by a decrease in DOCP until the strain reaches a value $\sim 0.5\,\%$ ($V_G \in [30, 90]\,\mathrm{V}$). An accurate explanation of this behaviour is hampered due to our inability to resolve the interplay of singlet and triplet bright trions that exhibit opposite polarization response (Fig. S7c,d; Ref. [33]). Furthermore, the strain-induced laser detuning effect is also expected to cause a drop in DOCP (see section section S7 for details). The DOCP increases again above 0.5 % strain as we approach the hybridization regime H2. Recent reports [33] have shown that the polarization response of X^-_{KK} intricately depends on intervalley scattering processes involving the

lower CB (Fig. S7e). These scattering processes change significantly in the hybridization regime through the lifted momentum-selection rules. Furthermore, the neutral intervalley $X_{KK'}$ and the spin-dark intravalley excitons X^{d}_{KK}, residing in the spectral vicinity of the bright trions ($\Delta E \sim 10\,\mathrm{meV}$), may have contributed to the observed increase in DOCP near 0.8 %. Similarly, the influence of KK–KQ hybridization on the valley polarization dynamics of X^{-}_{KK} (near 0.3 % strain) is not fully understood, warranting future studies.

S7 Potential artefacts in DOCP data

Laser detuning and cavity interference: Strain-induced bandgap reduction causes a strain-dependent shift in the detuning between the fixed excitation laser energy (1.81 or 1.84 eV) and the strain-dependent absorption energy of excitons. Therefore, our experiments exhibit a strain-dependent laser detuning effect:

$$\Delta E_{\mathrm{detune}}(\epsilon) = E_{\mathrm{laser}} - E_{X^{0}_{KK}}(\epsilon) \tag{S19}$$

Consequently, there is a strain-dependent decrease in the initial valley polarization (along with the associated DOCP and DOLP) of the KK excitons as the hot photoexcited carriers with strain-dependent excess energy undergo thermalization. Figure S8a demonstrates the laser detuning effect by showing measured DOLP vs. excitation energy in 1L-WSe_2 at $\epsilon = 0$. The DOLP decreases monotonically with energy detuning, which confirms that the reported DOCP in our experiments is an underestimation. The true amplification in the hybridization-related DOCP could be much higher compared to our experimental data with a fixed wavelength excitation laser. In the future, this DOCP could be probed with tunable wavelength exciton sources.

Additionally, the strain-dependent laser detuning may cause the excitation energy to become resonant with the A_{2s}/A_{3s} resonances at specific strain values. This may result in changes in light absorption, potentially influencing the valley polarization response. Furthermore, the cavity interference conditions, which change as a function of applied V_G, also influence the effective light absorption in the membrane and introduce nonlinear effects.

To investigate these artefacts, we repeated the DOCP measurements in device 2 under a CW excitation 1.96 eV (Fig. S8b), which is above the A_{2s}/A_{3s} resonance of 1L-WSe_2. Such an excitation also causes significantly different cavity interference conditions than the laser excitation at 1.84 eV (Fig. 2 in the main manuscript) and at 1.81 eV (Fig. S6c). While the absolute magnitude of DOCP under 1.96 eV excitation is, as expected, lower compared to the 1.84 eV excitation, a qualitatively similar response in DOCP is observed, including a non-monotonic behaviour vs. strain and a local DOCP maximum in the hybridization regime H2. These observations confirm that the laser

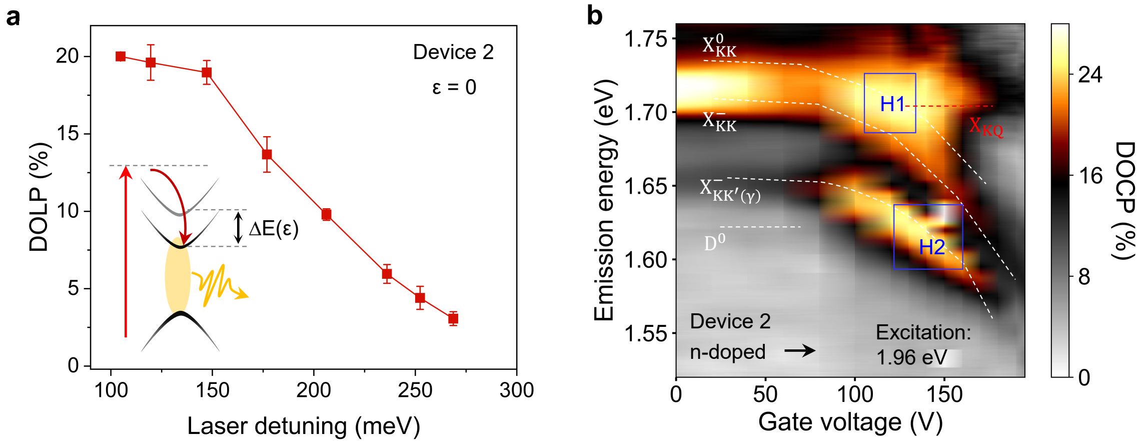

FIG. S8. **Laser detuning effect on polarization response. a** DOLP of X^{0}_{KK} vs. laser detuning energy, achieved by varying the excitation energy using a tunable pulsed laser source (coherent Chameleon Ultra-II + OPO-vis with a time-averaged power of 5 µW). The inset cartoon depicts the laser detuning effect, which is quantified via Eq. (S19) at $\epsilon = 0$. The DOLP decreases with increasing excitation energy, confirming the influence of laser detuning on exciton valley polarization. **b** False color map of DOCP vs. V_G in device 2 under 1.96 eV excitation. A 120 meV increase in the excitation energy (with respect to the data in Fig. S6b) is equivalent to nearly 1 % strain. Nevertheless, the DOCP response is qualitatively similar, with local maxima observed in both hybridization regimes H1 and H2.

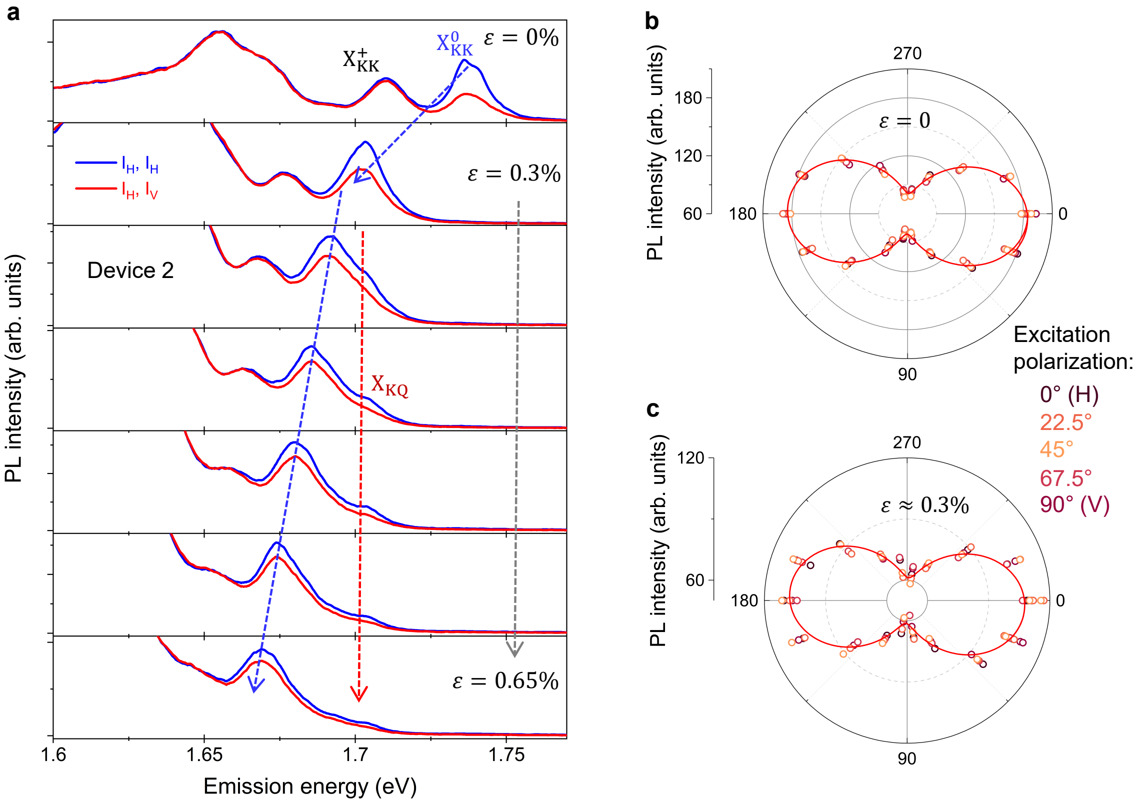

FIG. S9. **DOLP response of X^0_{KK} under KK–KQ hybridization.** **a** Co- (blue) and cross-linear (red) polarized PL line-cuts at selected strain values from device 2. Only X^0_{KK} retains linear polarization, consistent with the formation of a coherent state across K and K' valley [35]. The X_{KQ} state appears above 0.3 % strain, and remains near strain independent, consistent with the KK–KQ hybridization picture [1]. Critically, the hybridized state of KK–KQ excitons retains linear polarization, suggesting their coherently-coupled nature. Note, this device is p-doped, while the data in the main manuscript (Fig. 3b) corresponds to the same device being n-doped. Similarly, the DOCP data in Figs. 3a and 3c are from n- and p-doped devices, respectively. This confirms that while doping may alter the absolute magnitude of DOLP, the KK–KQ hybridization is characterized by an enhanced polarization response. **b,c** PL intensity of X^0_{KK} vs. analyzer angle under different linearly-polarized excitation at $\epsilon = 0$ (b) and 0.3 % (c); solid lines are fits to the data at a selected excitation polarization. The polarization direction of the emitted photons doesn't change with strain, ruling out strain anisotropy within the laser spot. Note, the seemingly identical emission direction for all excitation polarizations is due to the HWP placed in the common path of both incident and reflected beams (see Note S1 for details).

detuning and cavity effects do not significantly influence the interpretation of the manuscript's data.

Strain inhomogeneity and polarization anisotropy: While the strain in the center of the membrane is biaxial, away from the center it becomes spatially inhomogeneous ($\epsilon(r{=}1\,\mu\text{m}) = 0.9{\cdot}\epsilon_{\text{center}}$) and acquires a non-zero shear component [1]. Spatial inhomogeneity may influence the polarization-resolved data in several ways.

First, spatial inhomogeneity may induce strain anisotropy, which may break in-plane symmetry of excitons [36] and cause misinterpretation of the polarization-resolved data. To investigate strain anisotropy effects, we recorded polarization-resolved PL of X^0_{KK} by rotating the excitation polarization at different strain values, $\epsilon = 0$ and 0.3 % in Figs. S9b and S9c, respectively. We find that the polarization direction of the emitted photons remains the same regardless of the applied strain value, confirming an isotropic nature of the strain configuration in the center of the membrane.

Second, strain inhomogeneity causes broadening of the excitonic features in PL, which limits our ability to distinguish the polarization response of closely lying states. The influence of this effect is reflected in the TRPL data (Fig. 2f in the main manuscript). There, the TRPL trace at 0.8 % strain (red) has a long-lived component with time constant $\sim 230\,\text{ps}$, which is qualitatively similar to that at zero strain. This suggests that not all the dark excitons became bright at that strain level. We suggest that spatial inhomogeneity induced spectral overlap among several excitonic states (e.g., different phonon replicas of dark trions and charged biexcitons), thereby preventing the hybridization of

all dark states at a given strain level while the laser spot captured their dynamics.

Notably, the strain inhomogeneity becomes advantageous in identifying the hybridized state of X^0_{KK} and X_{KQ}, as described by our microscopic modeling (Note S2) and recent results [1]. The excitonic features related to this hybridization appear on the high energy tail in the PL spectrum, hence, are more sensitive to the device regions with local strain level around 0.35 %. This is also apparent from the increasing asymmetry in the linewidth of X^0_{KK} peak with strain (Fig. S9a), which leads to nonzero DOCP on the higher energy tail of the hybridized X^0_{KK} and X_{KQ} excitons (Fig. S6).

S8 Extended discussion on TRKR data

Probing at the excitonic energy: Since the excitonic energies are strain-dependent, to ensure that the TRKR dynamics were probed at desired strain levels and excitonic resonances, we recorded *in-situ* PL in the center of the membrane at each applied V_G during the TRKR measurements. Consequently, we tuned the pump/probe energies (FWHM ∼8 meV) across the X^0_{KK} and $X^{+/-}_{KK}$ resonances to optimize the TRKR signal.

Laser-induced heating: Suspended devices typically exhibit long thermalization time scales, exceeding 100 ns, which can influence optical responses through heating-induced shifts in excitonic resonances and other nonlinear effects. In our TRKR traces, we observe a finite, constant signal at negative delays for all applied gate voltages, which is consistent with heating-induced shifts in excitonic resonances and confirms that the thermalization time scale exceeds our laser pulse duration (∼13 ns). However, the timescale associated with our TRKR measurements, ∼100 ps, is three orders of magnitude smaller than the thermalization timescale; hence, the laser-heating of the membrane can be treated as a quasi-equilibrium state. This confirms that any changes in the TRKR signal at zero time delay arise due to pump-induced spin/valley polarization. To minimize the heating-induced nonlinear effects in our measurements, we maintained low pump/probe fluence around 3–6 µJ cm^{-2} throughout our experiments, corresponding to an injected exciton density on the order of 10^{11} cm^{-2}, significantly below the nonlinear regime.

Doping contributions to TRKR measurements: Doping changes, inherent to our straining technique, may influence the spin/valley dynamics in TMDs. Previous works have shown that TRKR probes near the trion resonance exhibit prolonged spin/valley lifetimes compared to the neutral exciton resonance [37–40]. These dynamics reflect the resident carrier polarization, showing a characteristic dependence on the injected carrier density [37–40]. To distinguish the carrier density- and strain-related effects, we examined the TRKR dynamics near exciton and trion resonances in devices with varying built-in carrier densities under two conditions:

i) carrier density was varied ($\Delta n_e \sim 4 \cdot 10^{11}$ cm^{-2}) with only minimal changes in strain level ($\Delta\epsilon < 0.05$ %, Fig. S10), and

ii) strain was applied together with carrier density changes in two devices: device 4 (n-doped, Fig. S11) and device 2 (p-doped, same as in the main manuscript; Fig. S12).

In the first case, only weak changes were observed in the spin/valley dynamics probed near neutral exciton resonance (Figs. S10b,d). We do not observe any long-lived component associated with either the neutral exciton or the trion resonance despite a carrier density change of $n_e \approx 4 \cdot 10^{11}$ cm^{-2} (Figs. S10d,e).

Conversely, in the second case, the long-lived component emerged in both devices when the applied strain level reached 0.3 %, and the TRKR dynamics were probed near the exciton resonance (Figs. S11b, S12b). The amplitude of the long-lived component decreased significantly as the strain level increased to ∼0.5 % (Fig S12e), eventually vanishing at 0.7 % strain (Fig. 4e in the main manuscript). In contrast, the TRKR dynamics near the trion resonance remained mono-exponential at all applied strain values in both devices, with time constants below 5 ps (Figs. S11c, S12c,f). Note, the two devices exhibit different doping levels in this strain regime: device 2 was slightly p-doped ($n_h \approx 1 \cdot 10^{11}$ cm^{-2}), whereas device 4 was n-doped, $n_e \approx 5.5 \cdot 10^{11}$ cm^{-2}.

These observations are in strong contrast to several reports where the long-lived TRKR dynamics were associated with trions, with significant dependence on the doping level [37, 38, 40]. We also note that long spin/valley lifetimes in those reports were critically influenced by substrate-related disorders [39] and excitons bound to trap states [38, 40, 41], that often cause a flip in the transient valley polarization [38] or a transition between mono- and bi-exponential decay profiles [39]. Our suspended devices are free of substrate-related disorder. Moreover, elevated sample temperature in our measurements facilitates the thermalization of shallow trap potentials and spatially varying charge puddles (Fig. 4b in the main manuscript). These observations rule out the doping-related effects as the cause of the long-lived TRKR component observed near the exciton resonance, suggesting an independent origin for the observed effect.

Our findings with a long-lived TRKR component in restricted strain range are in agreement with the formation of a partially intervalley state under KK–KQ hybridization. In contrast to the depolarization dynamics at zero strain, the exchange-driven valley depolarization gets suppressed for the hybridized intervalley states. Suppression of intervalley exchange coupling in the presence of a momentum-dark KQ exciton was theoretically predicted in Ref. [8], although this study did not account for the hybridization effects. We note that interpreting the dynamics above 0.7 % strain is challenging as the hybridization with the defects may alter the spin/valley depolarization channels. Here, distinguishing the spectral components related to excitons and trions becomes difficult due to the convolved PL spectrum at elevated temperatures. Nevertheless, the expected increase in the oscillator strength of dark excitons opens a new possibility to probe their spin/valley response via absorption-based methods. Systematic temperature- and strain-dependent measurements may be beneficial to disentangle these effects.

Strain inhomogeneity: In our TRKR measurements, we used a pump spot with a diameter of ∼3 µm, while the probe spot was tightly focused in the center of the membrane with a spot diameter of ∼1 µm. Although the pump-induced photoexcitation is spatially uniform within the probe region, strain inhomogeneity could facilitate exciton drift-diffusion dynamics, leading to time-dependent changes in the exciton population within the probe laser spot. This may influence the time-dependent signal probed in the center of the membrane. However, previous experiments have shown the drift length scale to be on the order of ∼10 nm on the time scale of 100 ps [26, 42], ruling out the possibility that the long-lived TRKR component is associated with exciton drift-diffusion dynamics.

We observe that the second decay component in the TRKR dynamics, albeit with weak amplitude, persists until ∼0.5 % strain (Fig. S12e). This suggests that the finite spot size of the probe laser may be sampling regions that are influenced by small inhomogeneities in the strain profile within the probe laser spot [1].

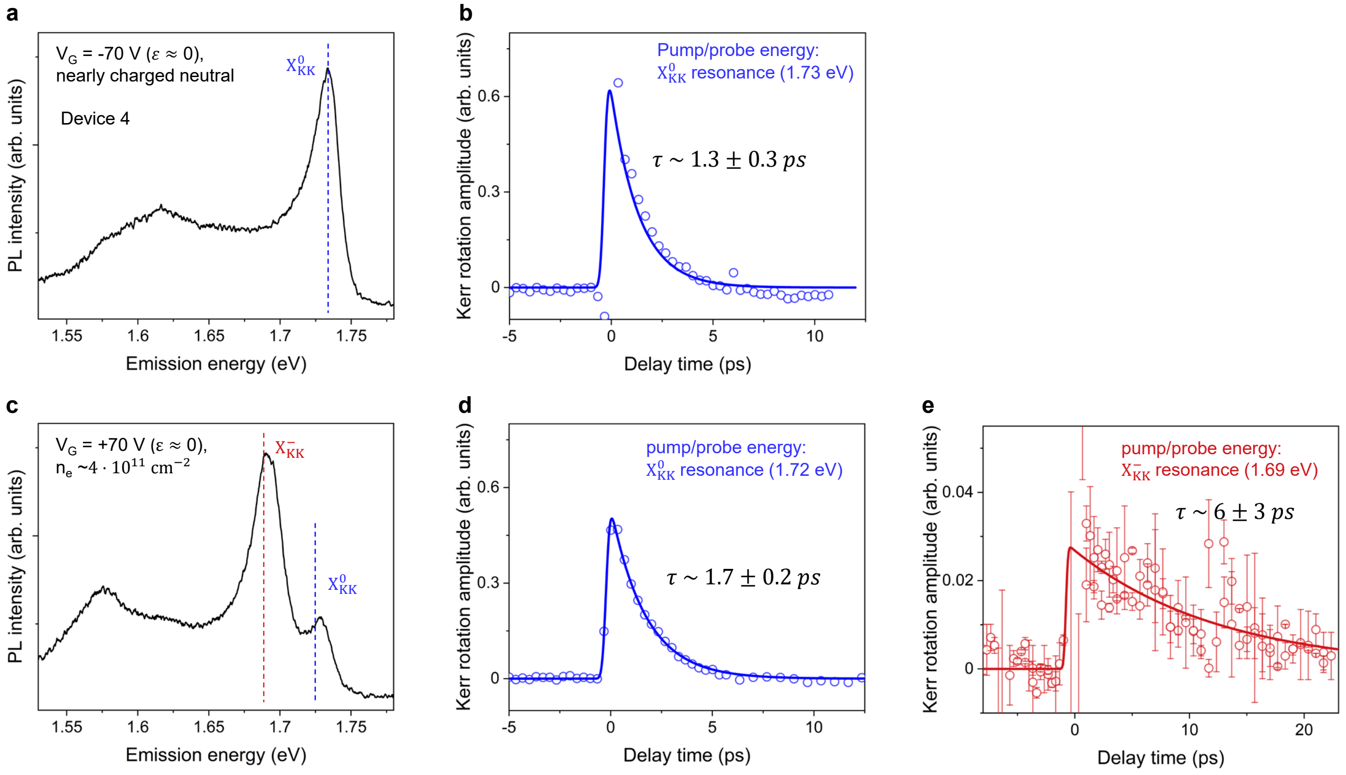

FIG. S10. **TRKR traces in weak strain regime at T = 100 K. a** PL spectrum in device 4 (measured *in-situ* under a pulsed excitation at 1.91 eV) to determine doping level and excitonic resonances during TRKR measurements. The device is nearly charged-neutral at V_G = -70 V. **b** TRKR dynamics probed near X^0_{KK} resonance are short-lived; corresponding pump/probe energies are denoted by the dashed line in a. No prominent TRKR signal is observed when probed below the exciton resonance. **c-e** PL from the same device at V_G = +70 V (c); the injected electron density n_e is $\sim 4 \cdot 10^{11}$ cm^{-2} while changes in the strain level are negligible. Corresponding TRKR dynamics probed near X^0_{KK} resonance (d) do not show any significant changes. A weak TRKR signal is observed near X^-_{KK} resonance (e), showing a qualitatively similar decay profile with ∼6 ps time constant.

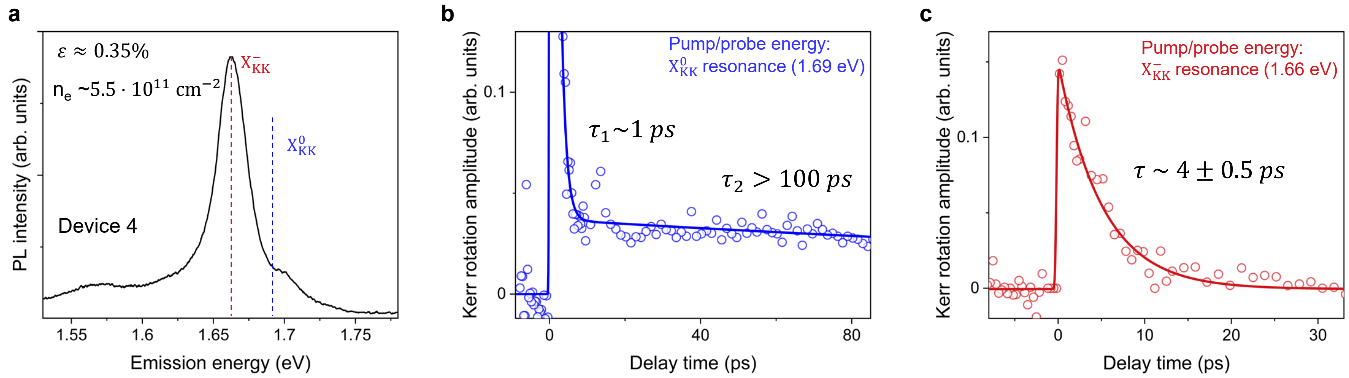

FIG. S11. **TRKR traces in device 4.** **a** PL spectrum at $\epsilon \approx 0.35\,\%$ ($V_G = +100\,V$). The dashed blue (red) line corresponds to TRKR probes near the exciton (trion) resonance, respectively. **b** TRKR dynamics probed near the neutral exciton resonance X^0_{KK}. A second, long-lived decay component emerges, similar to the data in Fig. 4 in the main manuscript. **c** TRKR dynamics near the trion resonance X^-_{KK} shows a mono-exponential decay profile with $\sim$4 ps time constant.

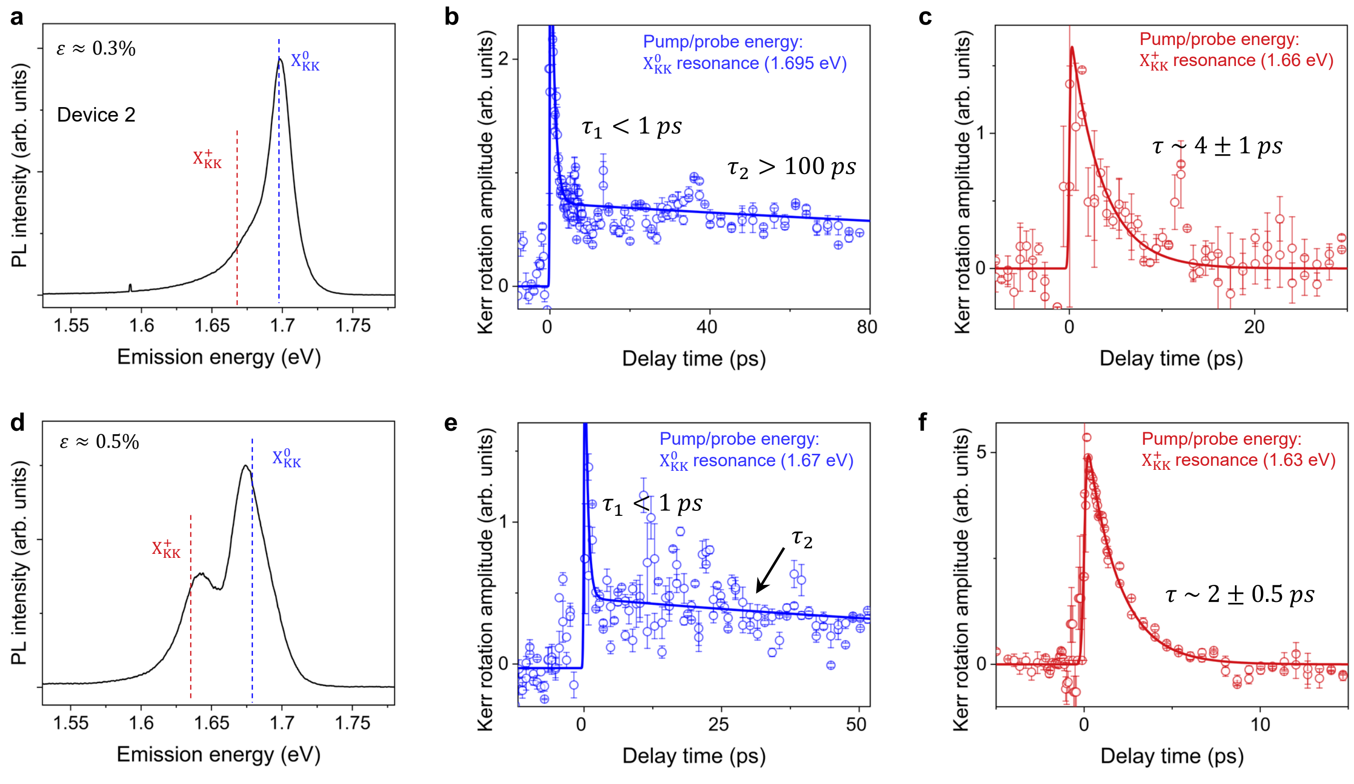

FIG. S12. **TRKR traces in device 2 at different strain levels.** **a** PL spectrum in device 2 (same as in Fig. 4 of the main manuscript) near $\epsilon \approx 0.3\,\%$, measured *in-situ* under a CW excitation at 1.96 eV. **b-c** Corresponding TRKR traces probed near X^0_{KK} (b) and X^+_{KK} resonances (c). **d-f** PL and TRKR traces probed near X^0_{KK} and X^+_{KK} resonances near 0.5% strain. A second decay component, albeit with significantly weaker amplitude, is observed only when probed near the higher energy transitions. The dynamics probed near trion resonances do not exhibit significant strain-dependent behaviour, confirming that the long-lived component is not related to the resident carrier polarization. Note that the apparent oscillatory features in some data sets are related to setup artefacts.

[1] Abhijeet M. Kumar, Denis Yagodkin, Roberto Rosati, Douglas J. Bock, Christoph Schattauer, Sarah Tobisch, Joakim Hagel, Bianca Höfer, Jan N. Kirchhof, Pablo Hernández López, Kenneth Burfeindt, Sebastian Heeg, Cornelius Gahl, Florian Libisch, Ermin Malic, and Kirill I. Bolotin. Strain fingerprinting of exciton valley character in 2D semiconductors. *Nature Communications*, 15(1):7546, August 2024.

[2] Roberto Rosati, Samuel Brem, Raül Perea-Causín, Robert Schmidt, Iris Niehues, Steffen Michaelis de Vasconcellos, Rudolf Bratschitsch, and Ermin Malic. Strain-dependent exciton diffusion in transition metal dichalcogenides. *2D Materials*, 8(1):015030, December 2020. Publisher: IOP Publishing.

[3] N S Rytova. The screening potential of a point charge in a thin film. *Proc. MSU, Phys., Astron.*, 3:30, 1967.

[4] L. Keldysh. Coulomb interaction in thin semiconductor and semimetal films. *JETP Letters*, 29:658, June 1979.

[5] Andor Kormányos, Guido Burkard, Martin Gmitra, Jaroslav Fabian, Viktor Zólyomi, Neil D. Drummond, and Vladimir Fal'ko. k·p theory for two-dimensional transition metal dichalcogenide semiconductors. *2D Materials*, 2(2):022001, April 2015. Publisher: IOP Publishing.

[6] Zahra Khatibi, Maja Feierabend, Malte Selig, Samuel Brem, Christopher Linderälv, Paul Erhart, and Ermin Malic. Impact of strain on the excitonic linewidth in transition metal dichalcogenides. *2D Materials*, 6(1):015015, November 2018.

[7] Klaus Zollner, Paulo E. Faria Junior, and Jaroslav Fabian. Strain-tunable orbital, spin-orbit, and optical properties of monolayer transition-metal dichalcogenides. *Physical Review B*, 100(19):195126, November 2019.

[8] Malte Selig, Florian Katsch, Samuel Brem, Garnik F. Mkrtchian, Ermin Malic, and Andreas Knorr. Suppression of intervalley exchange coupling in the presence of momentum-dark states in transition metal dichalcogenides. *Physical Review Research*, 2(2):023322, June 2020.

[9] Hongyi Yu, Gui-Bin Liu, Pu Gong, Xiaodong Xu, and Wang Yao. Dirac cones and Dirac saddle points of bright excitons in monolayer transition metal dichalcogenides. *Nature Communications*, 5(1):3876, May 2014. Publisher: Nature Publishing Group.

[10] Samuel Brem, Jonas Zipfel, Malte Selig, Archana Raja, Lutz Waldecker, Jonas D. Ziegler, Takashi Taniguchi, Kenji Watanabe, Alexey Chernikov, and Ermin Malic. Intrinsic lifetime of higher excitonic states in tungsten diselenide monolayers. *Nanoscale*, 11(25):12381–12387, 2019.

[11] Raul Perea-Causin, Daniel Erkensten, Jamie M. Fitzgerald, Joshua J. P. Thompson, Roberto Rosati, Samuel Brem, and Ermin Malic. Exciton optics, dynamics, and transport in atomically thin semiconductors. *APL Materials*, 10(10):100701, October 2022.

[12] K. Kośmider, J. W. González, and J. Fernández-Rossier. Large spin splitting in the conduction band of transition metal dichalcogenide monolayers. *Physical Review B*, 88(24):245436, December 2013. Publisher: American Physical Society.

[13] Filip Anselm Rasmussen and Kristian Sommer Thygesen. Computational 2D Materials Database: Electronic Structure of Transition-Metal Dichalcogenides and Oxides. *The Journal of Physical Chemistry Part C*, 119(23):13169–13183, 2015.

[14] Fedele Tagarelli, Edoardo Lopriore, Daniel Erkensten, Raül Perea-Causín, Samuel Brem, Joakim Hagel, Zhe Sun, Gabriele Pasquale, Kenji Watanabe, Takashi Taniguchi, Ermin Malic, and Andras Kis. Electrical control of hybrid exciton transport in a van der Waals heterostructure. *Nature Photonics*, 17(7):615–621, July 2023. Publisher: Nature Publishing Group.

[15] Ortwin Hess and Tilmann Kuhn. Maxwell-Bloch equations for spatially inhomogeneous semiconductor lasers. I. Theoretical formulation. *Physical Review A*, 54(4):3347–3359, October 1996. Publisher: American Physical Society.

[16] Yueh-Chun Wu, Takashi Taniguchi, Kenji Watanabe, and Jun Yan. Enhancement of exciton valley polarization in monolayer Mo S 2 induced by scattering. *Physical Review B*, 104(12):L121408, September 2021.

[17] M. Z. Maialle, E. A. De Andrada E Silva, and L. J. Sham. Exciton spin dynamics in quantum wells. *Physical Review B*, 47(23):15776–15788, June 1993.

[18] Marvin Kulig, Jonas Zipfel, Philipp Nagler, Sofia Blanter, Christian Schüller, Tobias Korn, Nicola Paradiso, Mikhail M. Glazov, and Alexey Chernikov. Exciton Diffusion and Halo Effects in Monolayer Semiconductors. *Physical Review Letters*, 120(20):207401, May 2018.

[19] F. Cadiz, C. Robert, E. Courtade, M. Manca, L. Martinelli, T. Taniguchi, K. Watanabe, T. Amand, A. C. H. Rowe, D. Paget, B. Urbaszek, and X. Marie. Exciton diffusion in WSe2 monolayers embedded in a van der Waals heterostructure. *Applied Physics Letters*, 112(15):152106, April 2018.

[20] Roberto Rosati, Raül Perea-Causín, Samuel Brem, and Ermin Malic. Negative effective excitonic diffusion in monolayer transition metal dichalcogenides. *Nanoscale*, 12(1):356–363, December 2019. Publisher: The Royal Society of Chemistry.

[21] Samuel Brem, August Ekman, Dominik Christiansen, Florian Katsch, Malte Selig, Cedric Robert, Xavier Marie, Bernhard Urbaszek, Andreas Knorr, and Ermin Malic. Phonon-Assisted Photoluminescence from Indirect Excitons in Monolayers of Transition-Metal Dichalcogenides. *Nano Letters*, 20(4):2849–2856, April 2020. Publisher: American Chemical Society.

[22] Iris Niehues, Robert Schmidt, Matthias Drüppel, Philipp Marauhn, Dominik Christiansen, Malte Selig, Gunnar Berghäuser, Daniel Wigger, Robert Schneider, Lisa Braasch, Rouven Koch, Andres Castellanos-Gomez, Tilmann Kuhn, Andreas Knorr, Ermin Malic, Michael Rohlfing, Steffen Michaelis de Vasconcellos, and Rudolf Bratschitsch. Strain Control of Exciton–Phonon Coupling in Atomically Thin Semiconductors. *Nano Letters*, 18(3):1751–1757, March 2018. Publisher: American Chemical Society.

[23] Roberto Rosati, Samuel Brem, Raül Perea-Causín, Robert Schmidt, Iris Niehues, Steffen Michaelis De Vasconcellos, Rudolf Bratschitsch, and Ermin Malic. Strain-dependent exciton diffusion in transition metal dichalcogenides. *2D Materials*, 8(1):015030, January 2021.

[24] Florian Dirnberger, Jonas D. Ziegler, Paulo E. Faria Junior, Rezlind Bushati, Takashi Taniguchi, Kenji Watanabe, Jaroslav

Fabian, Dominique Bougeard, Alexey Chernikov, and Vinod M. Menon. Quasi-1D exciton channels in strain-engineered 2D materials. *Science Advances*, 7(44):eabj3066, October 2021. Publisher: American Association for the Advancement of Science.

[25] Zhenghe Jin, Xiaodong Li, Jeffrey T. Mullen, and Ki Wook Kim. Intrinsic transport properties of electrons and holes in monolayer transition-metal dichalcogenides. *Physical Review B*, 90(4):045422, July 2014.

[26] Roberto Rosati, Robert Schmidt, Samuel Brem, Raül Perea-Causín, Iris Niehues, Johannes Kern, Johann A. Preuß, Robert Schneider, Steffen Michaelis De Vasconcellos, Rudolf Bratschitsch, and Ermin Malic. Dark exciton anti-funneling in atomically thin semiconductors. *Nature Communications*, 12(1):7221, December 2021.

[27] F. Libisch, S. Rotter, J. Güttinger, C. Stampfer, and J. Burgdörfer. Transition to Landau levels in graphene quantum dots. *Physical Review B*, 81(24):245411, June 2010. Publisher: American Physical Society.

[28] Pasqual Rivera, Minhao He, Bumho Kim, Song Liu, Carmen Rubio-Verdú, Hyowon Moon, Lukas Mennel, Daniel A. Rhodes, Hongyi Yu, Takashi Taniguchi, Kenji Watanabe, Jiaqiang Yan, David G. Mandrus, Hanan Dery, Abhay Pasupathy, Dirk Englund, James Hone, Wang Yao, and Xiaodong Xu. Intrinsic donor-bound excitons in ultraclean monolayer semiconductors. *Nature Communications*, 12(1):871, February 2021. Publisher: Nature Publishing Group.

[29] Kyrylo Greben, Sonakshi Arora, Moshe G. Harats, and Kirill I. Bolotin. Intrinsic and Extrinsic Defect-Related Excitons in TMDCs. *Nano Letters*, 20(4):2544–2550, April 2020. Publisher: American Chemical Society.

[30] Yaqian Wang, Longjiang Deng, Qilin Wei, Yi Wan, Zhen Liu, Xiao Lu, Yue Li, Lei Bi, Li Zhang, Haipeng Lu, Haiyan Chen, Peiheng Zhou, Linbo Zhang, Yingchun Cheng, Xiaoxu Zhao, Yu Ye, Wei Huang, Stephen John Pennycook, Kian Ping Loh, and Bo Peng. Spin-Valley Locking Effect in Defect States of Monolayer MoS2. *Nano Letters*, 20(3):2129–2136, March 2020. Publisher: American Chemical Society.

[31] Koloman Wagner, Edith Wietek, Jonas D. Ziegler, Marina A. Semina, Takashi Taniguchi, Kenji Watanabe, Jonas Zipfel, Mikhail M. Glazov, and Alexey Chernikov. Autoionization and Dressing of Excited Excitons by Free Carriers in Monolayer WSe2. *Physical Review Letters*, 125(26):267401, December 2020. Publisher: American Physical Society.

[32] Minhao He, Pasqual Rivera, Dinh Van Tuan, Nathan P. Wilson, Min Yang, Takashi Taniguchi, Kenji Watanabe, Jiaqiang Yan, David G. Mandrus, Hongyi Yu, Hanan Dery, Wang Yao, and Xiaodong Xu. Valley phonons and exciton complexes in a monolayer semiconductor. *Nature Communications*, 11(1):618, January 2020. Publisher: Nature Publishing Group.

[33] Cedric Robert, Sangjun Park, Fabian Cadiz, Laurent Lombez, Lei Ren, Hans Tornatzky, Alistair Rowe, Daniel Paget, Fausto Sirotti, Min Yang, Dinh Van Tuan, Takashi Taniguchi, Bernhard Urbaszek, Kenji Watanabe, Thierry Amand, Hanan Dery, and Xavier Marie. Spin/valley pumping of resident electrons in WSe2 and WS2 monolayers. *Nature Communications*, 12(1):5455, September 2021. Publisher: Nature Publishing Group.

[34] Robert Shreiner, Kai Hao, Amy Butcher, and Alexander A. High. Electrically controllable chirality in a nanophotonic interface with a two-dimensional semiconductor. *Nature Photonics*, 16(4):330–336, April 2022. Publisher: Nature Publishing Group.

[35] Aaron M. Jones, Hongyi Yu, Nirmal J. Ghimire, Sanfeng Wu, Grant Aivazian, Jason S. Ross, Bo Zhao, Jiaqiang Yan, David G. Mandrus, Di Xiao, Wang Yao, and Xiaodong Xu. Optical generation of excitonic valley coherence in monolayer WSe2. *Nature Nanotechnology*, 8(9):634–638, September 2013. Publisher: Nature Publishing Group.

[36] Denis Yagodkin, Kenneth Burfeindt, Zakhar A. Iakovlev, Abhijeet M. Kumar, Adrián Dewambrechies, Oguzhan Yücel, Bianca Höfer, Cornelius Gahl, Mikhail M. Glazov, and Kirill I. Bolotin. Excitons under large pseudomagnetic fields, December 2024. arXiv:2412.16596 [cond-mat].

[37] Luyi Yang, Nikolai A. Sinitsyn, Weibing Chen, Jiangtan Yuan, Jing Zhang, Jun Lou, and Scott A. Crooker. Long-lived nanosecond spin relaxation and spin coherence of electrons in monolayer MoS2 and WS2. *Nature Physics*, 11(10):830–834, October 2015.

[38] Manfred Ersfeld, Frank Volmer, Lars Rathmann, Luca Kotewitz, Maximilian Heithoff, Mark Lohmann, Bowen Yang, Kenji Watanabe, Takashi Taniguchi, Ludwig Bartels, Jing Shi, Christoph Stampfer, and Bernd Beschoten. Unveiling Valley Lifetimes of Free Charge Carriers in Monolayer WSe_2. *Nano Letters*, 20(5):3147–3154, May 2020.

[39] Jing Li, M. Goryca, K. Yumigeta, H. Li, S. Tongay, and S. A. Crooker. Valley relaxation of resident electrons and holes in a monolayer semiconductor: Dependence on carrier density and the role of substrate-induced disorder. *Physical Review Materials*, 5(4):044001, April 2021. Publisher: American Physical Society.

[40] Wei-Ting Hsu, Yen-Lun Chen, Chang-Hsiao Chen, Pang-Shiuan Liu, Tuo-Hung Hou, Lain-Jong Li, and Wen-Hao Chang. Optically initialized robust valley-polarized holes in monolayer WSe2. *Nature Communications*, 6(1):8963, November 2015.

[41] Xinlin Song, Saien Xie, Kibum Kang, Jiwoong Park, and Vanessa Sih. Long-Lived Hole Spin/Valley Polarization Probed by Kerr Rotation in Monolayer WSe_2. *Nano Letters*, 16(8):5010–5014, August 2016.

[42] Moshe G. Harats, Jan N. Kirchhof, Mengxiong Qiao, Kyrylo Greben, and Kirill I. Bolotin. Dynamics and efficient conversion of excitons to trions in non-uniformly strained monolayer WS2. *Nature Photonics*, 14(5):324–329, May 2020.
